# If Electric Cars Are Good for Reducing Emissions, They Could Be Even Better with Electric Roads

**DOI:** 10.1021/acs.est.2c00018

**Published:** 2022-06-23

**Authors:** Johannes Morfeldt, Wasim Shoman, Daniel J. A. Johansson, Sonia Yeh, Sten Karlsson

**Affiliations:** Physical Resource Theory, Department of Space, Earth and Environment, Chalmers University of Technology, Maskingränd 2, SE-412 96 Gothenburg, Sweden

**Keywords:** prospective life cycle assessment, greenhouse gas emissions, battery capacity, battery electric vehicles, electric road system, carbon footprint

## Abstract

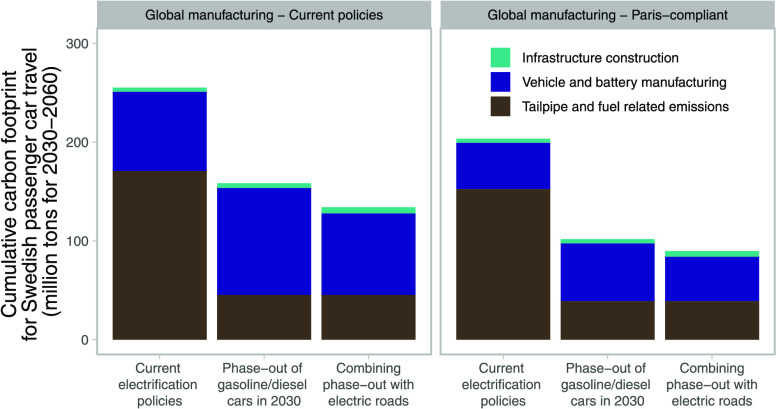

This research investigates carbon footprint impacts for full fleet electrification of Swedish passenger car travel in combination with different charging conditions, including electric road system (ERS) that enables dynamic on-road charging. The research applies a prospective life cycle analysis framework for estimating carbon footprints of vehicles, fuels, and infrastructure. The framework includes vehicle stock turnover modeling of fleet electrification and modeling of optimal battery capacity for different charging conditions based on Swedish real-world driving patterns. All new car sales are assumed to be electric after 2030 following phase-out policies for gasoline and diesel cars. Implementing ERS on selected high-traffic roads could yield significant avoided emissions in battery manufacturing compared to the additional emissions in ERS construction. ERS combined with stationary charging could enable additional reductions in the cumulative carbon footprint of about 12–24 million tons of CO_2_ over 30 years (2030–2060) compared to an electrified fleet only relying on stationary charging. The range depends on uncertainty in emission abatement in global manufacturing, where the lower is based on Paris Agreement compliance and the higher on current climate policies. A large share of the reduction could be achieved even if only a small share of the cars adopts the optimized battery capacities.

## Abstract 2

Implementing an electric road system could reduce the carbon footprint of passenger cars by enabling lower battery capacities compared to what is otherwise needed to achieve full electrification of the fleet.

## 1 Introduction

One of the main strategies for decarbonizing road transportation is the use of battery electric vehicles (BEVs) due to their high energy efficiency as well as zero tailpipe emissions.^1,2^ BEVs have low life cycle emissions when combined with low-carbon electricity supply.^3−7^ However, current passenger car users face many challenges when switching from internal combustion engine vehicles (ICEVs) to BEVs, including limited travel range, long charging time, and large investment costs due to high battery prices.^8^ Hence, relatively large batteries, which are cheap and charge fast, are needed to retain current travel behavior.^9^ The currently high battery prices are expected to drop over time,^10^ but concerns have been raised on the social and environmental sustainability of battery manufacturing.^11^ For example, current battery manufacturing is electricity-intensive and results in large greenhouse gas (GHG) emissions when situated in countries where electricity generation has not yet been decarbonized.^7,12^ Nevertheless, the Swedish Government^13^ has proposed a phase-out of gasoline and diesel passenger cars in new car sales from 2030 onwards, urging a faster pace of electrification. Electrification of road transportation is favorable in a country like Sweden since electricity generation is already largely decarbonized.^14^

The electric road system (ERS) technology allows for vehicles to be charged dynamically while driving, which could alleviate electrification barriers by increasing travel range and reducing charging times.^8,15^ Governmental agreements between Sweden and Germany are already initiated with the aim of intensifying cooperation on ERS research.^16^ Germany is considering overhead ERS technology that serves only heavy-duty vehicles,^17^ whereas Sweden is still testing technologies that could serve different vehicle types.^18^ The main driver for Sweden to implement the ERS technology is to promote the electrification of heavy-duty vehicles.^19^ Allowing for passenger cars to use such technology could enable additional benefits given that heavy-duty vehicles constitute only 4% of all vehicles in Sweden and contributed to 21% of tailpipe emissions in road transportation in 2019, whereas 94% of all vehicles are passenger cars and they contributed to 67% of tailpipe emissions.^20,21^

One of the main benefits of an ERS that also allows for charging by passenger cars is that it could enable downsizing of battery capacities for BEVs. Shoman et al.^9^ analyze real-world driving data and suggest that ERS could reduce battery sizes of BEVs by up to 75% and reduce the need for stationary charging, if not eliminating it at all for 30–70% of drivers depending on the ERS transfer power. Hence, an ERS that allows for passenger cars to charge could result in significant societal benefits and reduces costs compared to a system that only relies on stationary charging.^20,22^ Previous studies have shown significant GHG emission abatement potentials in enabling a larger part of the passenger car fleet to electrify through access to an ERS by estimating impacts on tailpipe emissions in Sweden and Norway,^23^ on tailpipe and fuel cycle emissions in the U.S.^15^ and on the life cycle, including tailpipe, infrastructure, battery manufacturing, and fuel cycle, in Washtenaw County in Michigan, U.S.^24^ While some studies have included a broad range of aspects of how an ERS could impact GHG emissions, none of the previous studies consider potential emission abatement measures in vehicle manufacturing and road construction supply chains.

The carbon footprint per kilometer of building and maintaining a road with an inductive ERS is around twice as high as building and maintaining a traditional road,^25^ but emissions related to road construction in Sweden could potentially decrease by 65% by 2030 compared to the current best available technology.^26^ Similarly, emissions related to battery manufacturing could potentially decrease by 24% by 2030 and 67% by 2050 if global manufacturing decarbonizes in line with the Paris agreement.^14^ These aspects have not yet been considered in previous studies nor in the context of full fleet electrification following phase-out policies for gasoline and diesel cars.

This research aims to bridge this gap by applying a prospective life cycle analysis framework that estimates the potential tradeoffs in emissions by implementing an ERS combined with stationary charging. The case study is based on Swedish passenger car travel under the assumption of phase-out policies for new gasoline and diesel cars, implemented as a ban in sales by 2030, and that current travel patterns are retained. The main contribution of this research is combining a battery and charging model based on real-world driving patterns with a vehicle stock turnover model and a carbon footprint estimation model. Hence, driving patterns at the level of individual car users in Sweden, which allows for more realistic estimates of battery size requirements, are combined with simulations of the future passenger car fleet. Ultimately, the research aims to answer the question: what are the carbon footprint impacts for passenger car travel of implementing ERS after phasing out gasoline and diesel cars?

## 2 Methodology

**Figure 1:**
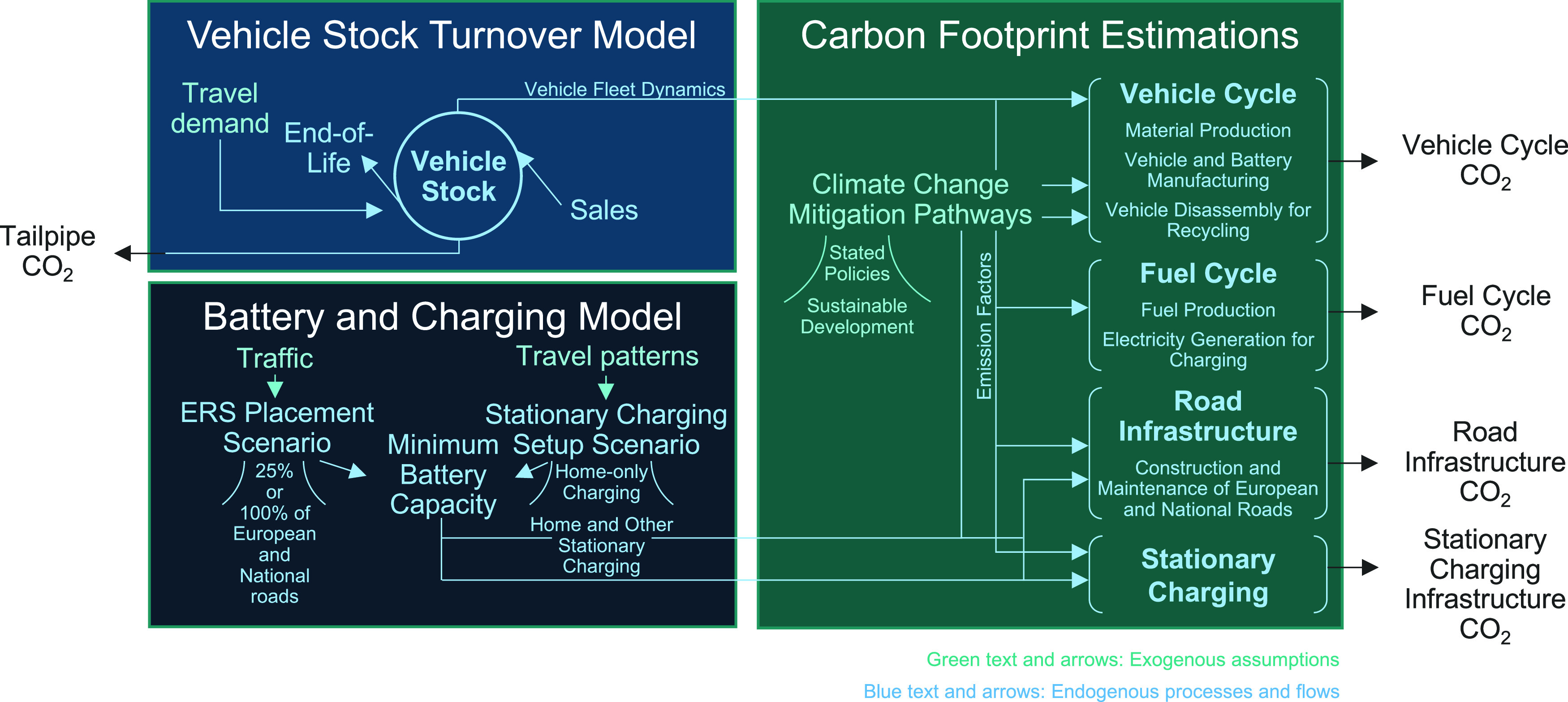
Analytical framework for the study.

The cumulative carbon footprint is estimated for the period 2030–2060, a time period equivalent to the average lifetime of an ERS.^15,23,27^ The estimation is based on the model Vehicle Turnover model Assessing Future Mobility services (V-TAFM).^14^ This model applies a prospective life cycle assessment framework for climate change impacts of Swedish passenger car travel with sensitivity analysis for different parameters. It is coupled with a model that estimates possible reductions in battery capacities using real-world driving patterns given assumed charging availability, i.e., stationary or dynamic on ERS, at the level of individual car users in Sweden.^9^ Together, the framework includes (i) vehicle stock turnover simulations (i.e., the electrification of the vehicle fleet), (ii) analyses of battery capacities needed given electric road placements in combination with available stationary charging infrastructure, and (iii) modeling of climate change mitigation pathways for global manufacturing (see Figure 1).

Several scenarios are analyzed to assess the impact of implementing ERS on the carbon footprint of Swedish passenger car travel in comparison to a current policy case without a phase-out of gasoline and diesel cars (see Table 1). This includes two scenarios for the extent of ERS placement (see details in Section 2.2.1), two scenarios for stationary charging (see details in Section 2.2.2), and two scenarios capturing the uncertainty in carbon footprint estimations (see details in Section 2.3). Moreover, several sensitivity cases are analyzed to highlight how the results are influenced by assumptions on the vehicle and battery lifetime, transfer power of the ERS, available battery sizes in the market, carbon footprint of the ERS technology and road construction, and adoption rate of BEVs with battery capacities optimized for using ERS (referred to as ERS-enabled BEVs).

**Table 1:** Scenario Setup and Main Assumptions^a^

	current policies (no phase-out of gasoline and diesel cars)	phase-out of gasoline and diesel cars with no ERS implementation	combining phase-out of gasoline and diesel cars with ERS implementation

stationary charging setup		home and other locations (or home-only locations in SI 2.3)	home and other locations (or home-only locations in SI 2.3)
ERS placement			25% of E&N roads (or 100% of E&N roads in SI 2.3)
vehicle stock turnover model	slow electrification (i.e., minimum EU requirements)	phase-out of new gasoline and diesel cars in 2030	phase-out of new gasoline and diesel cars in 2030
carbon footprint estimation	sustainable development or stated policies	sustainable development or stated policies	sustainable development or stated policies

a E&N roads: European and National Roads. SI: Supporting Information.

### 2.1 Vehicle Stock Turnover Model

The vehicle stock turnover model is used to capture the evolution of the Swedish passenger car fleet, including annual sales of new cars, annual number of cars reaching end-of-life, annual stock of cars, annual total vehicle energy use, and annual tailpipe CO_2_ emissions.^14^ The principle of the model is that the fleet needs to be large enough to meet an exogenous annual travel demand. New cars are added when old cars are retired, based on statistics on current vehicle lifetime^28^ in Sweden, and to meet the increasing annual travel demand. The lifetime of the battery is assumed to be equal to the vehicle lifetime (see reflections on this in Supporting Information, SI, 1.1) and a sensitivity analysis of this assumption is included to test its significance on the results. Life cycle CO_2_ emissions include tailpipe emissions, estimated based on the energy use of the fleet, as well as emissions related to the fuel cycle, vehicle cycle, and construction of infrastructure (see Section 2.3).

The assumed travel demand scenario is in line with the base prognosis by Swedish Transportation Administration.^29^ Fleet electrification is assumed in response to a phase-out of new gasoline and diesel cars in 2030, as proposed by the Swedish Government^13^ and in principle identical to the approach modeled by Morfeldt et al.^14^ A current policies scenario is included for comparison, where electrification of the fleet responds to the minimum requirements on tailpipe emissions set by the EU.^14,30^ The share of biofuel use is assumed in line with current biofuel policies^31^ until 2030 and thereafter kept constant throughout the modeling time horizon. The battery sizes of sold BEVs are optimized based on the available charging infrastructure in all scenarios, as described in Section 2.2. For scenarios with ERS implementation, cars sold before the construction of the ERS will remain in the fleet until retired and are gradually replaced with ERS-enabled BEVs. The adoption rate of ERS-enabled, ERS-enabled BEVs is assumed to be 100% from 2030 onwards in the main scenarios. A sensitivity analysis of this assumption is included to test the significance of different adoption rates on the results (see Section 3.3).

### 2.2 Battery and Charging Model

The battery and charging model assesses the charging infrastructure needs and the impact on BEVs’ battery requirements of implementing ERS based on real-world individual driving patterns for passenger cars in Sweden and a detailed geographic information system (GIS)-based infrastructure system. The model identifies the ERS utilization in different ERS placement scenarios and the potential reduction in battery capacities while fulfilling all driving requirements, according to each considered scenario.

We use a dataset that contains measurements of 716 private cars in Western Sweden^32^ but only select cars with at least 30 days of global positioning system (GPS) measurements for the analysis, resulting in 412 cars. The selected cars were randomly sampled from the Swedish vehicle registry with conditions on vehicle age of up to eight years and its registered owner’s age of up to 65 years. The survey was performed during 2010–2012 and covered all seasons. The dataset is considered representative of urban and rural areas in Western Sweden in terms of city size, household size, income and population density, car size, and fuel types.^32,33^ Additional reflections on the geographical representativeness of the data for Sweden on average are available in SI 1.2. The dataset has high temporal and spatial detail of the surveyed cars’ travel distance, visited locations, range limitations, utilized roads, parking areas/time, and home locations.

To identify charging occasions, this study applies a temporal approach to group trips based on parking lengths, as implemented in Shoman et al.^9^ In the main charging scenario (i.e., home and other stationary charging), stationary charging events occur when the parking time exceeds 4 h, which we identify as home (or near-home), and other charging points (e.g., public or work). For the home-only stationary charging scenario, stationary charging events occur when parking time exceeds 10 h, regardless of timing, or exceeds 8 h if the parking time includes 03:00 am.

All new BEVs on the market and sold in 2020 are assumed to be 3 times as energy-efficient as average, new ICEVs sold in 2020 in Sweden. The average specific energy use of new BEVs is also assumed to decrease by 10% until 2030, considering future energy efficiency improvements due to smaller transmission losses, higher energy density in batteries, and improvements in designing BEVs.^2,14^ While the impact of local road conditions, traffic, load, weather, etc., on specific energy use are not modeled explicitly, the assumption of new BEVs being 3 times more efficient than new ICEVs is expected to capture the impact of the average vehicle size in the Swedish fleet and how Swedish winter conditions have affected specific energy use of ICEVs. The average specific energy use of BEVs is assumed to decrease from *e* = 223 Wh/km in 2020 to *e* = 201 Wh/km in 2030 onwards. ERS could further contribute to reducing specific energy use due to reduced weight from a smaller battery size.^24^ Assuming constant average specific energy use over the year may however result in underestimated battery capacities in relation to the users’ required range during Swedish winter conditions given the large impact of ambient temperature on BEV range.^34^

#### 2.2.1 ERS Placement

Researchers generally propose installing ERS on selected roads with most traffic due to the large investment cost of implementing an ERS.^8,15,20,23,35−38^ European and National roads (E&N roads) constitute about 18,770 km (based on data for 2013), which is about 4% of the total road length in Sweden,^9,39^ while encompassing more than 50% of national freight and passenger traffic.^23^ The main scenario for ERS placement is 25% of E&N roads (equiv to 4690 km) in this study since it is considered to be the most economically beneficial.^9,23^ The roads are selected according to highest truck traffic^39^ since heavy-duty transport is expected to be the main motivation behind implementing an ERS in Sweden, as shown in Taljegard et al.^40^ A sensitivity case of implementing ERS on 100% of E&N roads highlights the impact of an extended ERS placement. In both cases, ERS is assumed to be installed on all lanes of the considered roads (equiv to a total of 13,561 km of ERS-lane for 25% of E&N roads and 40,123 km of ERS-lane for 100% of E&N roads).

The foreseen ERS is inductive, where electricity for charging is supplied via wireless power transfer from a coil in the road to a pick-up point in the vehicle.^23,41^ This technology has been tested at small scales, ranging from a test site of a few hundred meters to kilometers of public roads in several countries, e.g., Sweden, Germany, Japan, South Korea, and USA.^8,41^ The technology is anticipated to be commercially ready for deployment in the near future^42^ and is assumed to be ready for use in Sweden by 2030. This research assumes that the transfer power increases linearly with vehicle speed. The average transfer power is 2*e* (i.e., 2 times the assumed specific energy use of the vehicle), meaning that the battery’s state of charge is maintained and recharged by 1*e* while driving on ERS, assuming no transmission losses. The resulting average transfer power to the vehicle at 100 km/h on ERS would be 40 kW (assuming *e* = 201 Wh/km), which is an average assumption compared to the range of 20–50 kW considered in other studies.^9,15,43−46^ A sensitivity analysis of this assumption is included as well to test the significance of ERS transfer power (between 1*e* and 4*e*).

BEV users are assumed to charge their batteries whenever a charging opportunity is available—at stationary charging points to fully charge their batteries and while on an ERS charge their batteries with transfer power above *e*. For each scenario, the individual car’s minimum required battery capacity to fulfill all trips in its driving pattern is calculated and rounded up to the closest increment of 5 kWh. A sensitivity analysis of this assumption is included to test the significance of the availability of different battery sizes in the market.

#### 2.2.2 Stationary Charging Setup

Deployment of public stationary chargers is growing in Sweden.^47^ This indicates, together with the numerous support schemes for investments in public charging infrastructure initiated by the Swedish Government,^48−50^ that the network of public stationary chargers will be further extended as the fleet continues to electrify. Hence, our main scenario for stationary charging allows access to chargers at homes and in other public locations as required.

An alternative scenario with home-only stationary charging highlights the impact on the results of a restrictive deployment of public charging infrastructure. Chargeable cars are assumed to have access to a slow charger (i.e., power level of up to 7.4 kW; equivalent to Level 2 charging) at home (or near-home) in both scenarios. One home stationary charger is assumed to be deployed together with each chargeable car. The lifetime of a home charger is assumed to be about 8 years^51^ while the average vehicle and battery lifetime is assumed to be about 17 years^28^ (see SI 1.1 for reflections on the assumption on vehicle and battery lifetimes). Thus, the home chargers are assumed to be replaced once over the lifetime of the vehicle.

In the home and other stationary charging scenario, enough public chargers are assumed to be available to meet current EU regulations in addition to the home chargers, which require member states to have at least one publicly accessible charger per every 10 BEVs.^52^ Fast charging (i.e., power level ≥50 kW; equivalent to Level 3 charging) is assumed to be available at 15% of public chargers for users to keep their current refueling behaviors, whereas the remaining share is assumed to be slow chargers (i.e., power level of up to 22 kW; equivalent to Level 2 charging).^53−56^ Note that the battery size estimation only accounts for charging events of above 4 h. Hence, estimated battery sizes are not affected by extensive use of fast charging in the main case and instead captured in the sensitivity analysis that limits the available battery sizes in the market to 30–100 kWh.

Public stationary chargers are deployed in response to the growing fleet of chargeable cars. For simplicity, public chargers are deployed from 2020 onwards matching the current stock of chargeable cars. The number of chargers matches the future stock of chargeable cars when retired, based on the lifetime of each type of charger. This means that fast chargers are replaced to match the stock every 12 years from 2020 onwards, whereas slow chargers are replaced every 8 years.^51^

### 2.3 Carbon Footprint Estimations

The vehicle stock turnover model is linked to a carbon footprint estimation model (these models jointly create V-TAFM^14^). The carbon footprint estimation includes modeling of emerging technologies in the vehicle cycle (i.e., vehicle and battery manufacturing), and fuel cycle (i.e., production of liquid fuels and electricity used for charging). The vehicle types modeled are ICEVs, plug-in hybrid electric vehicles (PHEVs), and BEVs, and the fuels modeled are gasoline/diesel, biofuels, and electricity for charging (assumed to be average electricity from the Swedish grid). Liquid fuels and vehicles are assumed to be produced in global markets.

Two scenarios for carbon footprint estimations are constructed to capture the range of outcomes given the uncertainty in climate change mitigation efforts of global manufacturing (i.e., vehicles, liquid fuels, and stationary chargers). The two scenarios are based on future pathways for the carbon intensity of electricity modeled by the IEA^57^ and named Stated Policies and Sustainable Development. The former scenario is based on climate policies implemented or announced by governments in 2019, whereas the latter is designed to limit the global mean temperature increase to below 1.8 °C compared to the preindustrial level. In V-TAFM, additional modeling of emerging technologies in manufacturing processes is added to resemble the pathway of the two scenarios. The modeling is based on the GREET 2—Version 2019—LCA model,^58^ previous literature on emerging technologies, and logistic growth curves for emerging technologies (see details in Morfeldt et al.).^14^

In this study, the system boundary for estimating the carbon footprint of Swedish passenger car travel in V-TAFM is expanded to include the effects of ERS deployment. Hence, road and charging infrastructure has been added to the carbon footprint estimations as well as their construction and the required raw materials (see Figure 1). Emissions from construction and maintenance of all E&N roads are estimated for all scenarios and assumed to occur domestically. Hence, emissions factors for roads both with and without ERS developed by Balieu et al.^25^ are adjusted to account for Swedish industries reducing emissions in line with domestic climate policy targets as estimated by Karlsson et al.,^26^ see details in SI 1.3. A sensitivity analysis is included to test the significance of the assumption on emission factors for road construction decreasing in line with climate policy targets and to probe if higher emission factors for charging components instead would be used, based on Marmiroli et al.^59^ Stationary charging infrastructure is assumed to be manufactured in global markets. Hence, emission factors for different charger types are calculated based on material demand estimated by Zhang et al.^51^ and on the estimated carbon intensity of those materials in V-TAFM for the two climate change mitigation scenarios. Although emissions related to road and charging infrastructure should be attributed to all road transport vehicles, they are allocated fully to passenger cars in this research. The authors consider the ambiguity in assuming one allocation rule over the other and the subsequent risk of underestimating the carbon footprint of passenger cars to be too large.

Note that the low fuel cycle emissions for BEVs and PHEVs for the case of Sweden are made possible by the low carbon intensity of Swedish electricity generation and the assumed continued decarbonization of electricity generation in line with Swedish climate policy targets (see details in Morfeldt et al.).^14^

## 3 Results and Discussion

The results of estimating the cumulative carbon footprint for Swedish passenger car travel for the period of 2030–2060 (i.e., emissions from construction and maintenance of E&N roads with and without an ERS, for stationary chargers, tailpipe, vehicle, and fuel cycle emissions) are discussed below. The results highlight the main scenario of implementing an ERS with transfer power of 2*e* on 25% of E&N roads and allowing for home and other stationary charging. Annual results for tailpipe and fuel cycle emissions are provided in SI 2.1 and detailed results on the vehicle fleet dynamics are provided in SI 2.2. The results showing the sensitivity of these estimates to other assumptions on ERS coverage and placement, and restricted stationary charging are available in SI 2.3, on vehicle and battery lifetime in SI 2.4, on ERS transfer power in SI 2.5, on battery sizes available in the market in SI 2.6, and on higher emission factors for road construction and charging components in SI 2.7. Uncertainty in the carbon footprint estimations is indicated by the range between the two scenarios for emission abatement efforts in global manufacturing: Sustainable Development and Stated Policies.

### 3.1 Implementing an ERS Could Yield Significant Reductions in the Cumulative Carbon Footprint

**Figure 2:**
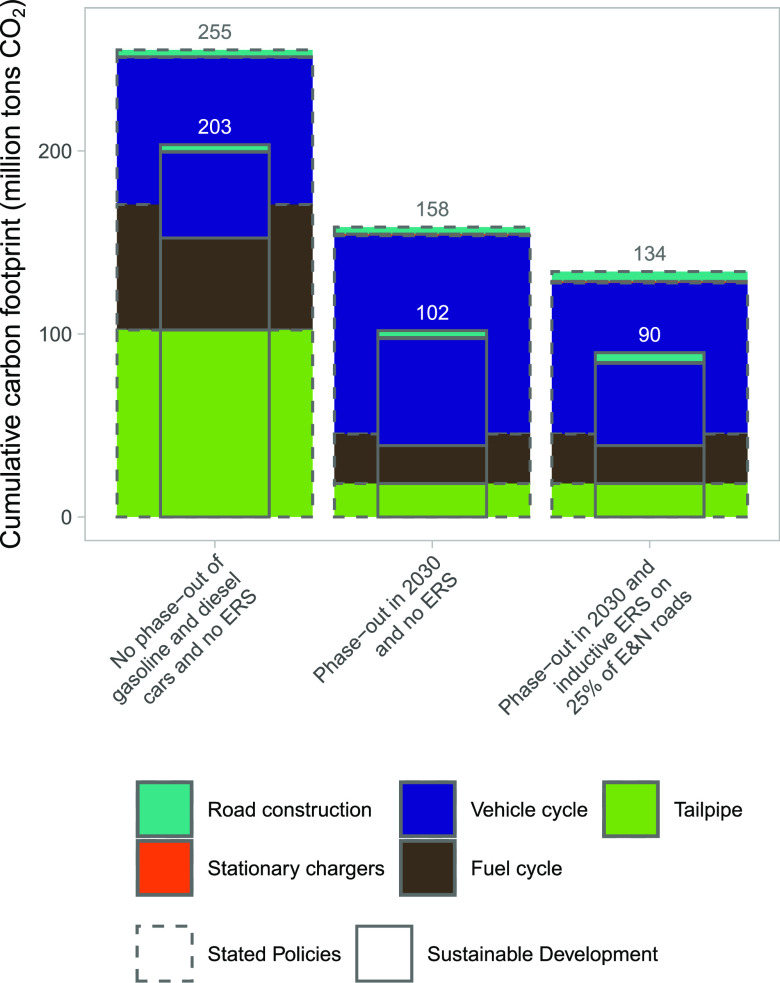
Cumulative carbon footprint of Swedish passenger car travel over the lifetime of an ERS, modeled for the period of 2030–2060. The phase-out is for new gasoline and diesel cars.

The cumulative carbon footprint for Swedish passenger car travel is estimated to be about 203–255 million tons of CO_2_ (MtCO_2_) over the period 2030–2060 for the current policies scenario without policies for phasing out gasoline and diesel cars (see Figure 2). When implementing such policies, the cumulative carbon footprint could decrease to about 102–158 MtCO_2_. The policies for phasing out gasoline and diesel cars are implemented as a ban in the model and result in 100% BEVs in new car sales by 2030 onwards, whereas the cumulative emissions could further decrease reaching levels of 90–134 MtCO_2_ if ERS is installed on 25% of E&N roads. The ranges indicate the uncertainty in emission abatement efforts made in global manufacturing (see results for Stated Policies vs. Sustainable Development in Figure 2). Hence, the emission abatement potential over the analyzed period is about 100 MtCO_2_ for phasing out gasoline and diesel cars, and an additional 12 and 24 MtCO_2_ if combined with ERS implementation for the Sustainable Development and Stated Policies pathways, respectively.

Expectedly, the potential benefits of an ERS for passenger cars are greater in cases where the carbon intensity of battery manufacturing is higher (i.e., when global manufacturing follows the Stated Policies pathway) since the effect of ERS on cumulative emissions for BEVs is through reducing required battery sizes. Emissions from vehicle and battery manufacturing alone become dominant in scenarios phasing out gasoline and diesel cars, reaching a share of 58–68% of the cumulative carbon footprint compared with 23–31% in the current policies scenario, in which the emissions largely originate from the tailpipe and the production of the fuels (i.e., the fuel cycle). In the case with a phase-out of gasoline and diesel cars and with ERS available, the cumulative emissions from vehicle and battery manufacturing constitute 50–62% of the total cumulative carbon footprint.

Extending ERS to cover 100% of E&N roads would result in a minor increase of the emissions reduction potential (ceteris paribus) by 4 MtCO_2_ for the Stated Policies scenario while the increase would be insignificant for Sustainable Development scenario (see SI 2.3). Thus, the additional decrease in battery sizes enabled by access to ERS on more roads is counteracted by additional emissions in ERS construction and maintenance. The low additional benefits of extending an ERS beyond high-traffic roads are in line with previous findings for the cases of the U.S.^15^ and Sweden.^23^ Even though an economic analysis is considered out of the scope of this study, it should be noted that the economic benefit for passenger cars of implementing ERS on 100% of E&N roads is also estimated to be low compared to 25% of E&N roads.^9^ Further, only allowing home charging in addition to the charging on the ERS has a negligible impact on the results (see SI 2.3). While the lifetime of the vehicle (and battery) would affect the pace of electrification—assuming a phase-out of gasoline and diesel cars in 2030, the estimated emission abatement potential for ERS implementation is still significant (see SI 2.4). The abatement potential significantly increases for a shorter lifetime assumption and decreases for longer lifetimes.

The technical specifications of a future ERS are still uncertain, including its transfer power.^44^ The results of a sensitivity analysis that tests the significance of the transfer power (between 1*e* and 4*e*) show that the reduction in the cumulative carbon footprint would be lower but still significant if the ERS transfer power is equal to the specific energy use of the vehicle (9–19 MtCO_2_ for 1*e* compared to 12–24 MtCO_2_ for 2*e*) (see SI 2.5). Hence, the results of the main case remain valid as long as the technology at least matches the vehicle energy use. The results also show that the marginal benefit of increasing the transfer power beyond 2*e* is small (reductions in the cumulative carbon footprint for 4*e* are estimated to 13–26 MtCO_2_).

The carbon footprints for both road infrastructure and stationary chargers are relatively small compared to the total carbon footprint. The cumulative carbon footprint of road construction and maintenance increases from 3.8 MtCO_2_ to reach 5.4 MtCO_2_ when implementing an ERS on 25% of E&N roads (see SI 2.3). These emissions increase further to 8.4 MtCO_2_ when ERS coverage is extended to 100% of E&N roads. The cumulative carbon footprint of home chargers is estimated to 0.4–0.6 MtCO_2_. In case public and other chargers are also considered, additional emissions of 0.1–0.3 MtCO_2_ are added to the cumulative carbon footprint. Note that ranges depend on global climate change mitigation pathways.

The uncertainty of emission abatement efforts in global manufacturing is not considered for road construction and maintenance-related emissions since they are assumed to occur domestically. Nevertheless, a sensitivity analysis highlights the impact on the results if Swedish road construction and maintenance does not decarbonize, effectively missing the domestic policy target, and that the emission factor for charging component would be larger (see SI 2.7). The cumulative carbon footprint of road construction and maintenance would then be 10.7 MtCO_2_ without an ERS and 16.2 and 26.9 MtCO_2_ for implementation on 25 and 100% of E&N, respectively. This means that the emission reduction potential of implementing an ERS would be slightly lower (8–20 MtCO_2_ for an ERS on 25% of E&N roads compared to 12–24 MtCO_2_ in the main case). Furthermore, extending ERS coverage from 25 to 100% of E&N roads would increase the cumulative carbon footprint compared to slightly reducing the cumulative emissions when assuming that road construction and maintenance are in line with the domestic climate policy targets. Note that these emissions are fully allocated to passenger cars to avoid using allocation rules even though roads and charging infrastructure would be shared with heavy-duty vehicles and public transportation.

### Implementing an ERS Could Yield Significant Avoided Emissions in Battery Manufacturing

**Figure 3:**
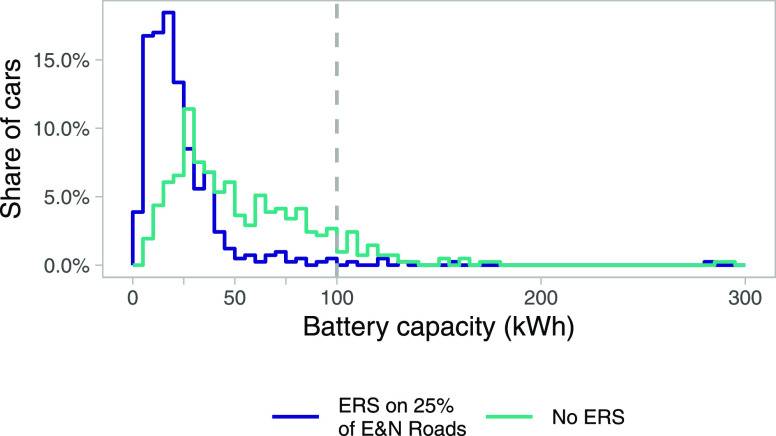
Share of cars with specific battery capacities, assuming post-2030 average specific energy use.

**Figure 4:**
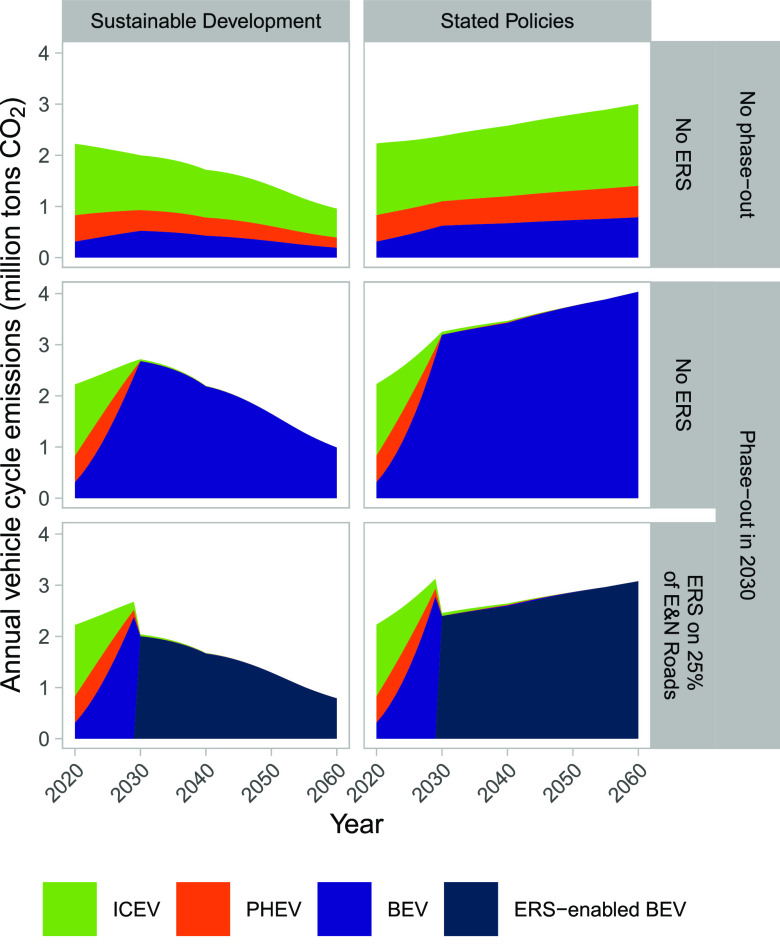
Annual vehicle cycle emissions, including manufacturing of batteries. The phase-out is for new gasoline and diesel cars.

High vehicle cycle emissions are currently attributed to BEVs with large battery sizes. Implementing ERS could result in significant avoided emissions by optimizing battery capacities in BEVs for the ERS (see Figure 3). This would result in reducing the battery capacities needed from 57 to 26 kWh for BEVs on average when using the ERS implemented on 25% of E&N roads (see Figure 4). The decrease in average battery sizes when implementing an ERS is partly due to a shift from a high share (i.e., 44%) of cars with battery sizes of over 50 kWh to a low share (i.e., 6% with ERS on 25% of E&N roads). With an ERS only 1% of battery sizes are above 100 kWh (i.e., the approximate battery size for current Tesla model S). The performed sensitivity analyses show the same pattern—implementing ERS could result in a significant shift toward smaller battery sizes (see Table 2). Detailed results for placement of ERS (25 or 100% of E&N roads), stationary charging (home and other places or home-only), ERS transfer power (1*e*–4*e*) are available in SI 2.5 and for limited availability of some battery sizes in the market in SI 2.6.

**Table 2:** Results in Terms of Average Battery Size and Share of Battery Sizes over 50 kWh for the Sensitivity Analyses^a^

		with ERS: average battery size (kWh)	with ERS: share of cars with battery sizes over 50 kWh (%)	without ERS: average battery size (kWh)	without ERS: share of cars with battery sizes over 50 kWh (%)
placement	100% of E&N roads	18	1	57	44
	25% of E&N roads	26	6	57	44
stationary charging	home-only	28	7	64	53
	home and other locations	26	6	57	44
ERS transfer power	1*e*	32	12	57	44
	4*e*	24	6	57	44
battery sizes in the market	fixed size—1 kWh-steps	24	6	55	44
	fixed size—5 kWh-steps	26	6	57	44
	fixed size—40 kWh-steps	46	10	73	55
	margin of 10 kWh	34	10	65	55
	size within 30–100 kWh range	34	6	54	44

a If not otherwise stated, main assumptions apply of 25% ERS on E&N roads, home, and other stationary charging, ERS transfer power of 2E, and fixed battery sizes of 5 kWh-steps.

Annual vehicle cycle emissions (i.e., emissions in vehicle and battery manufacturing) are estimated to 2.2 MtCO_2_ in 2020 and could reach 1.0–3.0 MtCO_2_ in 2060 with the current policies scenario, depending on emissions reductions in global manufacturing (see Figure 4). A phase-out of gasoline and diesel cars would result in a high demand for batteries for BEVs, increasing annual vehicle cycle emissions to 2.7–3.3 MtCO_2_ in 2030 and then reach 1.0–4.0 MtCO_2_ by 2060, depending on the pathway in global manufacturing. However, annual emissions from 2030 onwards could be partly mitigated by implementing ERS since the smaller battery sizes needed when using the ERS reduce the demand for battery size. In such a scenario, annual vehicle cycle emissions would increase until the implementation of the ERS. Then it would reach 0.8–3.1 MtCO_2_ by 2060, depending on global manufacturing pathway. Note that Figure 4 describes emissions related to sales of new cars. Details on the vehicle fleet dynamics can be found in SI 2.2.

Another way to describe the impact of implementing ERS is to consider the annual avoided emissions in battery manufacturing (i.e., the difference between only phasing out gasoline and diesel cars, the middle row in Figure 4, and combining a phase-out with implementing ERS, the lower panels in Figure 4). The annual avoided emissions could be 0.7 MtCO_2_ in 2030 and then decrease over time if global manufacturing follows Sustainable Development pathways, reaching a level of 0.2 MtCO_2_ by 2060. In contrast, the potential annual avoided emissions from implementing an ERS are higher throughout the period when global manufacturing follows Stated Policies pathways, starting at 0.8 MtCO_2_ in 2030 and increasing to around 1.0 MtCO_2_ per year in 2060.

Note that these results assume that all users choose the lowest battery capacity matching their travel patterns when buying a new BEV. Hence, one should interpret these results as showing the maximum impact of an ERS if car owners optimize the battery size of their car according to their driving pattern.

### 3.3 Importance of Adopting Battery Sizes Optimized for ERS in the Estimated Carbon Footprint Reductions

**Figure 5:**
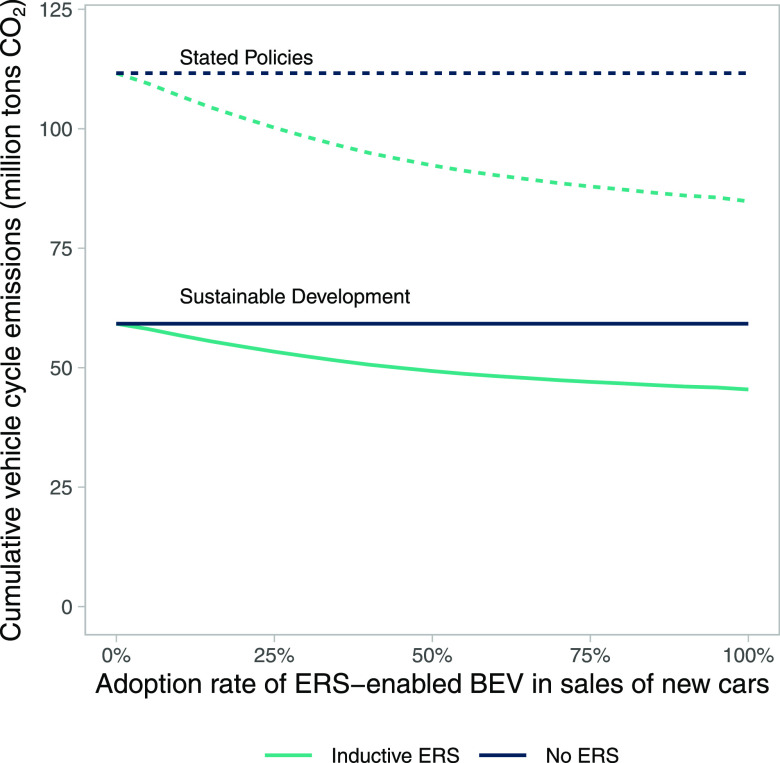
Cumulative vehicle cycle emissions for the period 2030–2060 depending on different adoption rates of ERS-enabled BEV as new car sales.

The emissions reduction potential of ERS is sensitive to the share of users that would buy an ERS-enabled car with battery optimized for their traveling needs as well as to the battery sizes available in the market at that time. The sensitivity analysis presented below explores how the cumulative vehicle cycle emissions for the period 2030–2060 are affected by the share of users buying an ERS-enabled car with optimal battery capacity (i.e., ERS-enabled BEVs) for their travel needs (see Figure 5).

The results from this sensitivity analysis assume that car users with the highest battery capacity reduction from buying ERS-enabled cars with battery sizes optimized for ERS would be the first adopters (see Figure 5). The remaining share of users are assumed to buy BEVs at the same capacity as they had prior to the ERS implementation. The results show that the reduction in emissions from battery manufacturing for adoption rates ranging from 0 to 50% (i.e., that 0–50% of new car sales are ERS-enabled BEVs) is more significant than when the adoption rate increases further up to 100%. The cumulative vehicle cycle emissions decrease by 8–17 MtCO_2_ with an ERS if those 50% of users with the largest savings adopt ERS-enabled BEVs compared to 12–24 MtCO_2_ with 100% adoption rate (see Figure 5). It is also worth noting that only around a tenth of users need to adopt ERS-enabled BEVs to reach breakeven in terms of emissions for implementing ERS on 25% of E&N roads (if higher emissions are assumed in construction (see SI 2.7), the share of users needed for breakeven increases to 20–35% depending on the pathway for global manufacturing).

Several additional sensitivity cases (see Table 2) related to the battery sizes that users would choose when buying a new BEV are also analyzed. Available battery size options for users to choose from may be limited in the future, which is tested in the sensitivity cases with fixed sizes of 1, 5, or 40 kWh. Range anxiety may discourage users from buying an optimal but small battery, which is tested in the sensitivity case that assumes a margin of 10 kWh, equivalent to about 50 km, on top of the optimal size suggested by the model. Finally, limited battery supply and intensified use of fast chargers for users with long-range needs may limit the upper boundary for battery sizes. Also, range anxiety among users could limit lower end for battery sizes. Both limits are tested in the last sensitivity case where only battery sizes within 30–100 kWh are considered. The results in terms of the cumulative carbon footprint are available in SI 2.6, showing that emissions reductions from ERS implementation could be significant also in these cases. Similar to the results in Figure 5, these reductions could be realized even if only users with the highest battery capacity reduction adopt ERS-enabled cars (see SI 2.8).

The distributions of required battery sizes for individual cars to meet each user’s travel demand show that a relatively high share of cars could manage with small battery sizes even without implementing an ERS (see Figure 3 and SI 2.5–2.6). These users would have small incentives in terms of reduced battery costs to adopt the slightly smaller battery capacity with the ERS. On the other hand, there is a share of users who would benefit highly from the potential reduction in battery sizes when using an ERS. These users could have significantly higher incentives for switching to ERS-enabled BEVs together with ERS access. However, there are other local circumstances that could influence the adoption rate of ERS-enabled BEVs, such as uncertain charging conditions that could induce range anxiety and subsequently encourage users to invest in larger battery sizes, and low cost–benefits of buying an ERS-enabled BEV. However, cost–benefit analyses are considered out of the scope of this study. Nevertheless, the sensitivity analyses suggest significant emissions reduction potential of implementing ERS even when the battery size options in the market are restricted or users choose a larger size than needed according to their travel patterns. The sensitivity analyses also show that not all users need to choose an optimum battery size for their traveling needs, but that the emissions reduction potentials are mainly dependent on those users with large potential battery size reductions (and the largest incentive to adopt a smaller battery).

Our analysis assumes that users are fully aware of their travel patterns and charging schedule to be able to minimize their need for battery sizes accordingly, even when their options are limited as in the sensitivity cases presented in SI 2.6. While this assumption can be motivated by the idea that users would seek to minimize investment costs when buying their BEVs, it is also likely that the decision to buy a certain battery size is influenced by other factors that are not taken into account in this study. However, the behavior of the users is assumed to be the same regardless of if they are buying an ERS-enabled car or not. Hence, the estimated reductions in battery sizes should not be considered as a forecast but as an estimate of possible emission savings. Furthermore, users are also assumed to be able to use the ERS without barriers including technical access to charging on the ERS, easy-to-use payment schemes, and pricing comparable to alternative stationary charging infrastructure.^9,15^ These conditions on ERS are still not certain.^15^

### 3.4 Policy Implications

The results of this study are considered valuable for policy discussions on public investment in charging infrastructure, including ERS and stationary chargers, and their related regulations. A Swedish public inquiry aims to evaluate and propose new regulations needed for implementing an ERS. The assignment for this inquiry has a strong focus on heavy transportation and includes aspects such as regulating access to the ERS and payment of use.^60^ It is accomplished in close collaboration with the newly appointed commission of electrification, whose goal is to promote the electrification of Swedish transportation in general.^61^

The most recent governmental assessment^19^ of a Swedish ERS implementation, which is one of the inputs to the public inquiry, only considers the benefits for heavy transportation. The assessment considers ERS implementation on certain selected high-traffic road segments, which is less extensive (3000 km) compared with our scenario of implementing an ERS on 25% of E&N roads (4690 km). The results show low additional benefits of an extended ERS placement beyond high-traffic road segments in terms of saved costs^9,23^ and additional emission reductions, which also have been implied by previous studies.^15,23^ Thus, these results are in accordance with the findings of this study. However, the above-mentioned assessment deems passenger car users’ interest in using an ERS to be low. This is partly due to the trend of increasing battery capacities in BEVs and partly due to barriers in terms of fees and business models that would be designed for businesses. In summary, the assessment considers the barriers to be too high for passenger car users to consider ERS as a charging possibility.

Nevertheless, our results show that implementing an ERS that serves passenger cars could enable significant additional emission reduction potentials. Also, our sensitivity analyses suggest that not all users would need to reduce their battery size to enable the benefits in terms of emissions reductions. Hence, a one-lane ERS that is the most probable case for an ERS for heavy transportation^23^ but provides more limited access for passenger cars, could still enable considerable benefits to passenger BEVs. Allowing passenger BEVs to charge on ERS could also enhance the societal benefits of the ERS in relation to its costs, which are considered to be low in the governmental assessment compared to the cost–benefits of increasing biofuel use for heavy transport.^19^ Furthermore, implementing an ERS could reduce the dependence on public stationary charging post-2030 without compromising the emission reduction potential of electrification according to our results. Hence, Swedish decision-makers could aim to reduce the barriers for passenger cars to use an implemented ERS to realize these benefits. An important step would be to propose regulations in a way that allows charging on ERS for both commercial vehicles and individually owned or leased cars.

While this study is limited to Sweden and mainly provides insights useful for Swedish decision-makers, the authors consider the methodology easily adapted to the contexts of other countries if similar data are available. Battery size reductions could also be achieved by a high density of fast chargers but that would be contingent on users’ tolerance to adjust their travel patterns to enable access to available chargers.^62^ While future, innovative battery technologies may allow for fast charging fully within minutes,^63^ current batteries require more time that can result in queuing—further inconveniencing the user—or over-dimensioned fast-charging infrastructure to cope with rush hour demands.^56^ Since this study considers a future where passenger car users are not restricted by charging infrastructure and retain their current travel patterns, thereby facilitating the transition toward electric passenger car travel, scenarios with comprehensive fast charging as an option for reducing battery sizes are disregarded. However, further investigating combinations of additional deployment of fast chargers with ERS could be an interesting direction for future research.

## Supporting Information Available

Additional methodological details—reflections on vehicle lifetime and on the geographical representativeness of data on travel behaviors as well as assumptions for the carbon footprint estimation of road and charging infrastructure—and detailed results: tailpipe and fuel cycle emissions, vehicle fleet dynamics, sensitivity analyses—home-only charging, extended coverage of ERS, ERS transfer power, battery sizes available in the market, higher emissions in road construction and maintenance, battery/vehicle lifetime, and adoption rate of low-batter-capacity BEVs (PDF)

## References

[ref1] de Coninck H.; Revi A.; Babiker M.; Bertoldi P.; Buckeridge M.; Cartwright A.; Araos Maldives M.; Bakker S.; Bazaz A.; Belfer E.; Benton T.; de Coninck H.; Revi A.; Babiker M.; Bertoldi P.; Buckeridge M.; Cartwright A.; Dong W.; Ford J.; Fuss S.; Hourcade J.; Ley D.; Mechler R.; Newman P.; Revokatova A.; Schultz S.; Steg L.; Sugiyama T.; Masson-Delmotte V.; Zhai P.; Pörtner H. O.; Roberts D.; Skea J.; Shukla P.; Pirani A.; Moufouma-Okia W.; Péan C.; Pidcock R.; Connors S.; R Matthews J. B.; Chen Y.; Zhou X.; Gomis M. I.; Lonnoy E.; Maycock T.; Tignor M.; Waterfield T. Strengthening and Implementing the Global Response. In Global warming of 1.5 °C. An IPCC Special Report on the Impacts of Global Warming of 1.5 °C above Pre-industrial Levels and Related Global Greenhouse Gas Emission Pathways, in the Context of Strengthening the Global Response to the Threat of Climate Change, Masson-Delmotte V.; Zhai P.; Pörtner H. O.; Roberts D.; Skea J.; Shukla P. R.; Pirani A.; Moufouma-Okia W.; Péan C.; Pidcock R.; Connors S.; Matthews J. B. R.; Chen Y.; Zhou X.; Gomis M. I.; Lonnoy E.; Maycock T.; Tignor M.; Waterfield T., Eds.; IPCC: Switzerland, 2018.

[ref2] MIT Energy Initiative. Insights into Future Mobility. Cambridge, MA, USA, 2019. http://energy.mit.edu/insightsintofuturemobility (accessed March 16, 2021).

[ref3] Kamiya G.; Axsen J.; Crawford C. Modeling the GHG Emissions Intensity of Plug-in Electric Vehicles Using Short-Term and Long-Term Perspectives. Transp. Res. D: Transp. Environ. 2019, 69, 209–223. 10.1016/j.trd.2019.01.027.

[ref4] Ellingsen L. A.-W.; Singh B.; Strømman A. H. The Size and Range Effect: Lifecycle Greenhouse Gas Emissions of Electric Vehicles. Environ. Res. Lett. 2016, 11, 054010 10.1088/1748-9326/11/5/054010.

[ref5] Hoekstra A. The Underestimated Potential of Battery Electric Vehicles to Reduce Emissions. Joule 2019, 3, 1412–1414. 10.1016/j.joule.2019.06.002.

[ref6] Wu Z.; Wang M.; Zheng J.; Sun X.; Zhao M.; Wang X. Life Cycle Greenhouse Gas Emission Reduction Potential of Battery Electric Vehicle. J. Cleaner Prod. 2018, 190, 462–470. 10.1016/j.jclepro.2018.04.036.

[ref7] Nordelöf A.; Messagie M.; Tillman A. M.; Ljunggren Söderman M.; Van Mierlo J. Environmental Impacts of Hybrid, Plug-in Hybrid, and Battery Electric Vehicles—What Can We Learn from Life Cycle Assessment?. Int. J. Life Cycle Assess 2014, 19, 1866–1890. 10.1007/s11367-014-0788-0.

[ref8] Domingues-Olavarría G.; Márquez-Fernández F. J.; Fyhr P.; Reinap A.; Alaküla M. Electric Roads: Analyzing the Societal Cost of Electrifying All Danish Road Transport. World Electr. Veh. J. 2018, 9, 1–11. 10.3390/wevj9010009.

[ref9] Shoman W.; Karlsson S.; Yeh S. Benefits of Including Battery Electric Cars in Electric Road Systems: Battery and Infrastructure Savings; Chalmers University of Technology: Gothenburg, Sweden, 2021. https://research.chalmers.se/publication/523472 (accessed March 16, 2021).

[ref10] Mongird K.; Fotedar V.; Viswanathan V.; Koritarov V.; Balducci P.; Hadjerioua B.; Alam J. Energy Storage Technology and Cost Characterization Report, PNNL-28866; Pacific Northwest National Laboratory, HydroWIRES, U.S. Department of Energy: Richland, Washington, US, 2019. https://www.pnnl.gov/publications/energy-storage-technology-and-cost-characterization-report (accessed March 16, 2021).

[ref11] International Energy Agency (IEA). Global EV Outlook 2019 - Scaling-up the Transition to Electric Mobility; OECD/IEA: Paris, France, 2019. https://www.iea.org/reports/global-ev-outlook-2019 (accessed March 16, 2021).

[ref12] Davidsson Kurland S. Energy Use for GWh-Scale Lithium-Ion Battery Production. Environ. Res. Commun. 2019, 2, 012001 10.1088/2515-7620/ab5e1e.

[ref13] Swedish Government. Regeringens Proposition 2019/20:65 En Samlad Politik För Klimatet – Klimatpolitisk Handlingsplan (Governmental Bill 2019/20:65 Joint Policy for Climate Change - Climate Policy Action Plan), 2020. https://www.regeringen.se/rattsliga-dokument/proposition/2019/12/prop.-20192065/ (accessed March 16, 2021).

[ref14] Morfeldt J.; Davidsson Kurland S.; Johansson D. J. A. Carbon Footprint Impacts of Banning Cars with Internal Combustion Engines. Transp. Res. D: Transp. Environ. 2021, 95, 102807 10.1016/j.trd.2021.102807.

[ref15] Limb B. J.; Asher Z. D.; Bradley T. H.; Sproul E.; Trinko D. A.; Crabb B.; Zane R.; Quinn J. C. Economic Viability and Environmental Impact of In-Motion Wireless Power Transfer. IEEE Trans. Transp. Electrif. 2019, 5, 135–146. 10.1109/TTE.2018.2876067.

[ref16] Jöhrens J.; Helms H.; Nebauer G.; Jelica D.In Feasibility Study of Swedish-German Corridor with Electric Road System, Annual Transport Conference at Aalborg University, 2020; pp 1–4.

[ref17] Kühnel S.; Hacker F.; Görz W. Oberleitungs-Lkw Im Kontext Weiterer Antriebs- Und Energieversorgungsoptionen Für Den Straßengüterfernverkehr; Öko-Institut e.V.: Berlin, Germany, 2018. https://www.oeko.de/fileadmin/oekodoc/StratON-O-Lkw-Technologievergleich-2018.pdf (accessed March 16, 2021).

[ref18] Nordin L. Life Cycle Assessments for Electric Road Systems; VTI PM: Linköping, Sweden, 2020. http://vti.diva-portal.org/smash/get/diva2:1444976/FULLTEXT01.pdf (accessed March 16, 2021).

[ref19] Swedish Transportation Administration. Regeringsuppdrag - Analysera Förutsättningar Och Planera För En Utbyggnad Av Elvägar (Government Task - Analyze Pre-Requisites and Plan for Implementation of Electric Roads), 2021. http://urn.kb.se/resolve?urn=urn:nbn:se:trafikverket:diva-4498 (accessed March 16, 2021).

[ref20] Fyhr P.; Domingues G.; Andersson M.; Marquez-Fernandez F. J.; Bangtsson H.; Alakula M. Electric Roads: Reducing the Societal Cost of Automotive Electrification. In 2017 IEEE Transportation Electrification Conference and Expo (ITEC); IEEE, 2017; pp 773–778 10.1109/ITEC.2017.7993367.

[ref21] Willerström J. Modelling CO2 Emissions from Passenger Cars for Swedish Municipalities; Uppsala University, 2019. http://urn.kb.se/resolve?urn=urn:nbn:se:uu:diva-385843 (accessed March 16, 2021).

[ref22] Marquez-Fernandez F. J.; Domingues-Olavarria G.; Lindgren L.; Alakula M. Electric Roads: The Importance of Sharing the Infrastructure among Different Vehicle Types. In 2017 IEEE Transportation Electrification Conference and Expo, Asia-Pacific (ITEC Asia-Pacific); IEEE, 2017; pp 1–6 10.1109/ITEC-AP.2017.8080780.

[ref23] Taljegard M.; Thorson L.; Odenberger M.; Johnsson F. Large-Scale Implementation of Electric Road Systems: Associated Costs and the Impact on CO 2 Emissions. Int. J. Sustainable Transp. 2020, 14, 606–619. 10.1080/15568318.2019.1595227.

[ref24] Bi Z.; Keoleian G. A.; Lin Z.; Moore M. R.; Chen K.; Song L.; Zhao Z. Life Cycle Assessment and Tempo-Spatial Optimization of Deploying Dynamic Wireless Charging Technology for Electric Cars. Transp. Res. C: Emerg. Technol. 2019, 100, 53–67. 10.1016/j.trc.2019.01.002.

[ref25] Balieu R.; Chen F.; Kringos N. Life Cycle Sustainability Assessment of Electrified Road Systems. Road Materials and Pavement Design 2019, 20, S19–S33. 10.1080/14680629.2019.1588771.

[ref26] Karlsson I.; Rootzén J.; Johnsson F. Reaching Net-Zero Carbon Emissions in Construction Supply Chains – Analysis of a Swedish Road Construction Project. Renewable Sustainable Energy Rev. 2020, 120, 109651 10.1016/j.rser.2019.109651. PMC7352113 34234615

[ref27] Viktoria Swedish ICT. Slide-in Electric Road System; Gothenburg, Sweden, 2013. http://urn.kb.se/resolve?urn=urn:nbn:se:ri:diva-26026 (accessed March 16, 2021).

[ref28] Transport Analysis. Detailed Excerpt on Scrappage of Vehicles from the Statistics “Fordon På Väg” (Road Vehicles) for the Years 2014-2018; Stockholm: Sweden, 2020. https://www.trafa.se/vagtrafik/fordon/ (accessed April 2, 2020).

[ref29] Swedish Transportation Administration. Prognos För Persontrafiken 2040 - Trafikverkets Basprognoser 2020-06-15 (Prognosis for Passenger Transportation 2040 - the Swedish Transportation Administration’s Base Prognosis 2020-06-14); Borlänge: Sweden, 2020. https://www.trafikverket.se/for-dig-i-branschen/Planera-och-utreda/Planerings--och-analysmetoder/Samhallsekonomisk-analys-och-trafikanalys/Kort-om-trafikprognoser/ (accessed March 16, 2021).

[ref30] Regulation (EU) 2019/631 of the European Parliament and of the Council of 17 April 2019 Setting CO2 Emission Performance Standards for New Passenger Cars and for New Light Commercial Vehicles, and Repealing Regulations (EC) No 443/2009 and (EU) No 510/201. Off. J. Eur. Union 2019, 62, 80.

[ref31] Swedish Government. Bränslebytet förstärks med högre inblandning av förnybart i drivmedel (The emissions reduction obligation quota policy is reinforced with increasing the share of renewables in vehicle fuels). https://www.regeringen.se/pressmeddelanden/2020/09/branslebytet-forstarks-med-hogre-inblandning-av-fornybart-i-drivmedel/ (accessed September 30, 2020).

[ref32] Karlsson S. The Swedish Car Movement Data Project - Final Report, PRT Report 2013:1, Rev 2; Chalmers University of Technology: Gothenburg, Sweden, 2013; https://research.chalmers.se/en/publication/187380 (accessed March 16, 2021).

[ref33] Jakobsson N.; Gnann T.; Plötz P.; Sprei F.; Karlsson S. Are Multi-Car Households Better Suited for Battery Electric Vehicles? - Driving Patterns and Economics in Sweden and Germany. Transp. Res. C: Emerg. Technol. 2016, 65, 1–15. 10.1016/j.trc.2016.01.018.

[ref34] Iora P.; Tribioli L. Effect of Ambient Temperature on Electric Vehicles’ Energy Consumption and Range: Model Definition and Sensitivity Analysis Based on Nissan Leaf Data. World Electr. Veh. J. 2019, 10, 2 10.3390/wevj10010002.

[ref35] Ranch P. Förstudie: Elektriska Vägar - Elektrifiering Av Tunga Vägtransporter (Pre-Study: Electric Roads - Eletrification of Heavy Road Transportation); Grontmij: Stockholm, Sweden, 2010; http://www.elvag.se/en/archive/2010-04-30/forstudie.pdf (accessed March 16, 2021).

[ref36] den Boer E.; Aarnink S.; Kleiner F.; Pagenkopf J. Zero Emissions Trucks: An Overview of State-of-the-Art Technologies and Their Potential; CE Delft: Delft, the Netherlands, 2013 https://www.cedelft.eu/publicatie/zero_emission_trucks/1399 (accessed March 16, 2021)..

[ref37] Musavi F.; Edington M.; Eberle W. Wireless Power Transfer: A Survey of EV Battery Charging Technologies. In 2012 IEEE Energy Conversion Congress and Exposition (ECCE); IEEE, 2012; pp 1804–1810 10.1109/ECCE.2012.6342593.

[ref38] Connolly D. ERoads - A Comparison between Oil, Battery Electric Vehicles, and Electric Roads for Danish Road Transport in Terms of Energy, Emissions, and Costs; Aalborg University: Aalborg, 2016. https://vbn.aau.dk/en/publications/eroads-a-comparison-between-oil-battery-electric-vehicles-and-ele (accessed March 16, 2021).

[ref39] Swedish Transport Administration. Lastkajen – Sveriges väg- och järnvägsdata (The loading dock - Swedish road and railway data) - Excerpt of data on the Swedish road network and traffic volumes for the year 2013. https://www.trafikverket.se/tjanster/data-kartor-och-geodatatjanster/hamta-var-oppna-data/lastkajen---sveriges-vag--och-jarnvagsdata/ (accessed May 1, 2020).

[ref40] Taljegard M.; Göransson L.; Odenberger M.; Johnsson F. Spacial and Dynamic Energy Demand of the E39 Highway – Implications on Electrification Options. Appl. Energy 2017, 195, 681–692. 10.1016/j.apenergy.2017.02.025.

[ref41] Chen F.; Taylor N.; Kringos N. Electrification of Roads: Opportunities and Challenges. Appl. Energy 2015, 150, 109–119. 10.1016/j.apenergy.2015.03.067.

[ref42] Chen Z.; Liu W.; Yin Y. Deployment of Stationary and Dynamic Charging Infrastructure for Electric Vehicles along Traffic Corridors. Transp. Res. C: Emerg. Technol. 2017, 77, 185–206. 10.1016/j.trc.2017.01.021.

[ref43] Lindgren L. Electrification of City Bus Traffic: - A Simulation Study Based on Data from Linköping; Lund Institute of Technology: Lund, Sweden, 2017. http://iea.lth.se/publications/Reports/LTH-IEA-7265.pdf (accessed March 16, 2021).

[ref44] García-Vázquez C. A.; Llorens-Iborra F.; Fernández-Ramírez L. M.; Sánchez-Sainz H.; Jurado F. Comparative Study of Dynamic Wireless Charging of Electric Vehicles in Motorway, Highway and Urban Stretches. Energy 2017, 137, 42–57. 10.1016/j.energy.2017.07.016.

[ref45] Jang Y. J. Survey of the Operation and System Study on Wireless Charging Electric Vehicle Systems. Transp. Res. C: Emerg. Technol. 2018, 95, 844–866. 10.1016/j.trc.2018.04.006.

[ref46] Siqi Li.; Mi C. C. Wireless Power Transfer for Electric Vehicle Applications. IEEE J. Emerging Sel. Top. Power Electron. 2015, 3, 4–17. 10.1109/JESTPE.2014.2319453.

[ref47] Power Circle. Laddinfrastrukturstatistik (Statistics on public charging infrastructure). https://www.elbilsstatistik.se/laddinfrastatistik (accessed March 8, 2021).

[ref48] Swedish Government. Förordning (2020:577) Om Statligt Stöd För Utbyggnad Av Publika Laddstationer För Snabbladdning Av Elfordon (Ordinance 2020:577 on Governmental Support for Extension of Public Charging Stations for Fast Charging of Electric Vehicles); Swedish Government, 2020. https://www.riksdagen.se/sv/dokument-lagar/dokument/svensk-forfattningssamling/forordning-2020577-om-statligt-stod-for_sfs-2020-577 (accessed March 16, 2021).

[ref49] Swedish Government. Förordning (2015:517) Om Stöd till Lokala Klimatinvesteringar (Ordinance 2015:517 on Support for Local Climate Investments); Swedish Government, 2015. https://www.riksdagen.se/sv/dokument-lagar/dokument/svensk-forfattningssamling/forordning-2015517-om-stod-till-lokala_sfs-2015-517 (accessed March 16, 2021).

[ref50] Swedish Government. Förordning (2019:525) Om Statligt Stöd För Installation Av Laddningspunkter För Elfordon (Ordinance 2019:525 on Governmental Support for Installation of Charging Points for Electric Vehicles); Swedish Government, 2019. https://www.riksdagen.se/sv/dokument-lagar/dokument/svensk-forfattningssamling/forordning-2019525-om-statligt-stod-for_sfs-2019-525 (accessed March 16, 2021).

[ref51] Zhang Z.; Sun X.; Ding N.; Yang J. Life Cycle Environmental Assessment of Charging Infrastructure for Electric Vehicles in China. J. Cleaner Prod. 2019, 227, 932–941. 10.1016/j.jclepro.2019.04.167.

[ref52] European Parliament and Council. Directive 2014/94/EU of the European Parliament and the Council of 22 October 2014 on the Deployment of Alternative Fuels Infrastructure, 2014.

[ref53] Amsterdam Roundtable Foundation; McKinsey&Company. Electric Vehicles in Europe: Gearing up for a New Phase?; McKinsey&Company, 2014. https://www.mckinsey.com/featured-insights/europe/electric-vehicles-in-europe-gearing-up-for-a-new-phase (accessed March 18, 2021).

[ref54] Chen T.; Zhang X. P.; Wang J.; Li J.; Wu C.; Hu M.; Bian H. A Review on Electric Vehicle Charging Infrastructure Development in the UK. Journal of Modern Power Systems and Clean Energy 2020, 8, 193–205. 10.35833/MPCE.2018.000374.

[ref55] Brazil R. Recharging the Future; Education in Chemistry, Royal Society of Chemistry, 2017. https://edu.rsc.org/feature/recharging-the-future/2500345.article (accessed March 9, 2022).

[ref56] Gnann T.; Funke S.; Jakobsson N.; Plötz P.; Sprei F.; Bennehag A. Fast Charging Infrastructure for Electric Vehicles: Today’s Situation and Future Needs. Transp. Res. D: Transp. Environ. 2018, 62, 314–329. 10.1016/j.trd.2018.03.004.

[ref57] International Energy Agency (IEA). World Energy Outlook 2019; OECD/IEA: Paris, France, 2019; iea.org/weo (accessed March 16, 2021).

[ref58] Argonne National Laboratory. The Greenhouse Gases, Regulated Emissions, and Energy Use in Transportation (GREET) Model - GREET 2, Version 2019. https://greet.es.anl.gov/ (accessed January 9, 2020).

[ref59] Marmiroli B.; Dotelli G.; Spessa E. Life Cycle Assessment of an On-Road Dynamic Charging Infrastructure. Appl. Sci. 2019, 9, 3117 10.3390/app9153117.

[ref60] Swedish Government. Kommittédirektiv - Elvägar, Dir. 2020:105 (Committee Directive - Electric Roads). 2021. https://www.regeringen.se/4a9366/contentassets/11134442747b47fdb1e6bc57dfd8a7e0/bilaga-kommittedirektiv-elvagar.pdf (accessed March 16, 2021).

[ref61] Swedish Government. Elektrifieringskommissionens Uppdrag (Directive for the Electrificafion Commission). 2020. https://www.regeringen.se/regeringens-politik/transportsektorn-elektrifieras/el-1/ (accessed March 16, 2021).

[ref62] Wood E.; Neubauer J. S.; Burton E. In Quantifying the Effect of Fast Charger Deployments on Electric Vehicle Utility and Travel Patterns via Advanced Simulation, SAE Technical Papers 2015-01-1687, 2015. 10.4271/2015-01-1687.

[ref63] Yang X. G.; Liu T.; Wang C. Y. Thermally Modulated Lithium Iron Phosphate Batteries for Mass-Market Electric Vehicles. Nat. Energy 2021, 6, 176–185. 10.1038/s41560-020-00757-7.

