# Supplementary material for: If Electric Cars Are Good for Reducing Emissions, They Could Be Even Better with Electric Roads
Source: Environ Sci Technol. 2022 Jun 23;56(13):9593–603. doi: 10.1021/acs.est.2c00018 (PMC9261191; doi:10.1021/acs.est.2c00018)
Supplement: Supplementary file 1 — es2c00018_si_001.pdf [file es2c00018_si_001.pdf]

If electric cars are good for reducing emissions, they could be even better with electric roads

Johannes Morfeldt[1,*], Wasim Shoman[1], Daniel J. A. Johansson[1], Sonia Yeh[1], Sten Karlsson[1]

[1] Physical Resource Theory, Department of Space, Earth and Environment, Chalmers University of Technology, Maskingränd 2, SE-412 96 Gothenburg, Sweden.

* Corresponding author: johannes.morfeldt@chalmers.se

Supporting Information

Table of Contents

1 Methodological details S2
1.1 Reflections on assumptions on vehicle lifetime S2
1.2 Reflections on assumptions on geographical representativeness of data on travel behaviors S2
1.3 Carbon footprint estimations of road and charging infrastructure S3
2 Detailed results S5
2.1 Tailpipe and fuel cycle emissions S5
2.2 Vehicle fleet dynamics S6
2.3 Sensitivity analysis – ERS on 100% of E&N roads and for home-only stationary charging S8
2.4 Sensitivity analysis – Vehicle and battery lifetime S9
2.5 Sensitivity analysis – ERS transfer power S10
2.6 Sensitivity analysis – assumptions on battery sizes available in the market S11
2.7 Sensitivity analysis – higher emissions in road construction and maintenance S12
2.8 Sensitivity analysis – importance of the adoption rate of ERS-enabled BEVs S13
References S14

Table of Figures

Figure SI 1: Cumulative share of individual drivers that drive less than a certain daily distance and cumulative share of distance covered by those drivers, for Swedish counties (*län*) ranked by population density (blue – low, and yellow – high). S3
Figure SI 2: Annual tailpipe emissions for Swedish passenger car travel. Note that this emission pathway is equal for all scenarios analyzed. S5
Figure SI 3: Annual fuel cycle emissions for Swedish passenger car travel. Note that these emissions depend on the assumed climate change mitigation pathway of global manufacturing S5
Figure SI 4: Annual sales of Swedish passenger cars for the analyzed drivetrains under the assumption of a ban on ICEs in 2030, depending on implementation of ERS. S6
Figure SI 5: Annual stock of cars (i.e., cars available in the fleet) available to supply Swedish passenger car travel demand for the analyzed drivetrains, depending on implementation of ERS. S6
Figure SI 6: Annual number of Swedish passenger cars reaching end-of-life for the analyzed drivetrains, depending on implementation of ERS. S7
Figure SI 7: Annual vehicle energy use per energy carrier for Swedish passenger car travel. Note that these pathways are equal for all scenarios analyzed meaning that vehicle energy use is assumed to be equal regardless of ERS implementation. S7
Figure SI 8: Cumulative carbon footprint of Swedish passenger car travel over the lifetime of an ERS for all scenarios compared to ERS placement on 100% of E&N roads, modelled for the period of 2030 – 2060. The ban is for gasoline and diesel cars. S8
Figure SI 9: Cumulative carbon footprint for infrastructure construction and maintenance for all scenarios compared to ERS placement on 100% of E&N roads, modelled for the period of 2030 – 2060. .... S8
Figure SI 10: Cumulative carbon footprint of Swedish passenger car travel over the lifetime of an ERS for all scenarios, modelled for the period of 2030 – 2060. The ban is for gasoline and diesel cars. Sensitivity cases with varying assumed vehicle and battery lifetime. ERS placement on 25% of E&N roads, home and other stationary charging, transfer power of 2e and fixed battery sizes (5 kWh-steps). S9
Figure SI 11: Share of cars with specific battery capacities, assuming post-2030 average specific energy use. Sensitivity cases with varying transfer power of 1e – 4e. S10
Figure SI 12: Cumulative carbon footprint of Swedish passenger car travel over the lifetime of an ERS for all scenarios, modelled for the period of 2030 – 2060. The ban is for gasoline and diesel cars. Sensitivity cases with varying transfer power of 1e – 4e. ERS placement on 25% of E&N roads, home and other stationary charging and fixed battery sizes (5 kWh-steps). S10
Figure SI 13: Share of cars with specific battery capacities, assuming post-2030 average specific energy use. Sensitivity cases with varying battery sizes available in the market. ERS transfer power of 2e. S11
Figure SI 14: Cumulative carbon footprint of Swedish passenger car travel over the lifetime of an ERS for all scenarios, modelled for the period of 2030 – 2060. The ban is for gasoline and diesel cars. Sensitivity cases with varying battery sizes available in the market. ERS placement on 25% of E&N roads, home and other stationary charging and ERS transfer power of 2e. S11
Figure SI 15: Cumulative carbon footprint over the lifetime of an ERS, modelled for the period of 2030 – 2060. The ban is for gasoline and diesel cars. Sensitivity case for higher emissions in road construction and maintenance. S12
Figure SI 16: Cumulative carbon footprint for charging and road infrastructure over the lifetime of an ERS, modelled for the period of 2030 – 2060. Sensitivity case for higher emissions in road construction and maintenance. S12
Figure SI 17: Cumulative vehicle cycle emissions for the period 2030-2060 depending on different adoption rates of ERS-enabled BEV as new car sales. Sensitivity cases with varying transfer power of 1e – 4e (columns) and varying battery sizes available in the market (rows). S13

1 Methodological details

1.1 Reflections on assumptions on vehicle lifetime

All vehicles are assumed to have a lifetime of around 17 years, implemented as a normal distribution calibrated to vehicle retirement statistics for 2014-2018[1]. The calibrated normal distribution has a mean of 16.93 and a standard deviation of 0.26 multiplied by 16.93. We assume that vehicle lifetimes will stay constant in the future and that the lifetime of a battery is as long as the vehicle. Previous research[2,3] has made similar assumptions motivated by batteries being charged at slow rates when using an ERS which allows for a long lifetime. At the same time, other studies suggest that the lifetime of batteries may be limited when increasing the number of charging and discharging cycles, especially with high depth-of-discharge rates[4]. This could happen if the car often leaves the ERS and almost fully discharges the battery before returning. The number of cycles would increase when using smaller battery capacities with an ERS as compared with using a larger battery with stationary charging. This suggests that the battery of a ERS-enabled BEV adopted for use with an ERS could have a shorter lifetime than a regular BEV. On the other hand, as argued by Bi et al.[5], a smaller battery capacity would result in a lighter car, which would reduce the driving energy use and result in less cycling of the battery for fulfilling the same travel demand. Finally, recent advances in the technical design of vehicle batteries could enable lifetimes much longer than the lifetime of the vehicle regardless of depth-of-discharge or number of charging and discharging cycles[6].

1.2 Reflections on assumptions on geographical representativeness of data on travel behaviors

The estimated emission reduction potentials of implementing an ERS are based on detailed travel patterns of 412 cars from Western Sweden (i.e., county of *Västra Götalands län* and *Kungsbacka* municipality). Thus, the study assumes that these travel patterns are representative of Sweden as a whole. The data is considered representative for the southern parts of Sweden but may be lacking representation of movement patterns in sparsely populated areas, such as rural areas in Northern Sweden[7]. This is confirmed by comparing data for different counties in the Swedish National Travel survey[8] showing that daily distances for individual drivers are similar for *Västra Götalands län* as compared to the Swedish average but differ slightly from counties with sparse population density, see discussion below. In such counties, longer trips make up a larger share of the total daily distance travelled on average, which may indicate a need for larger battery capacities in those counties compared to the sample. Hence, additional samples from different parts of Sweden could strengthen the analysis. Moreover, changes in travel behaviors are assumed to be negligible over time, and when switching to BEVs, despite the assumed increase in travel demand from 116 billion vehicle-kilometers in 2020 to 179 billion vehicle-kilometers in 2060, based on the prognosis[3] estimates for 2018, 2040 and 2065. The prognosis assumes an increase in population and income while the cost of driving is expected to decrease. Those factors may also influence travel behavior and movement patterns.

The detailed movement patterns used in the battery capacity model are based on a dataset with geographic representation of a county in Western Sweden (county of *Västra Götalands län* and *Kungsbacka* municipality)[7] and movement patterns are taken as representative for Sweden as a whole. In particular, the daily distance travelled per driver is of interest as a proxy for the case of home-only stationary charging if we assume that the majority of drivers in that case charges once per day. To assess how representative *Västra Götalands län* is of Sweden as a whole, Statistics from the Swedish National Travel survey has been aggregated to show the distribution for daily driving distances, see Figure SM 1.

The results for *Västra Götalands län* certainly are close to the national average, estimated in *All counties* in the figure, although slightly biased towards counties with higher population density (i.e., inhabitants per km^2). This can be explained by *Västra Götalands län* containing the second largest city of Sweden, even though it is considered a representative mix of rural and urban areas in Sweden[7]. However, the results for *Västra Götalands län* differ from counties with low population density. *Västra Götalands län* has a population density of 73 inhabitants per km^2, which is higher than *Norrbottens län* that represents the lowest in Sweden of 2.6 inhabitants per km^2 but lower than *Stockholms län*'s population density that represents the highest in Sweden with 367 inhabitants per km^2, all referring to the year 2020[9].

Given that *Västra Götalands län* is close to the national averages, the results based on the Battery Capacity Model, explained in SM 1.1 above, are considered accurate at the aggregated national level. However, care should be taken when discussing county-specific issues related to the battery capacities needed to cover the daily distance. For the counties with lower population densities, a larger share of the cumulative distance is covered by farther daily distances as compared to the national average or to *Västra Götalands län*, which indicates that a larger battery capacity may be needed for drivers in those counties. On the other hand, the opposite is observed for a few counties with higher population densities where more of the cumulative distance is covered by shorter daily distances. A more detailed analysis using data on movement patterns from a larger number of counties could therefore strengthen the analysis.

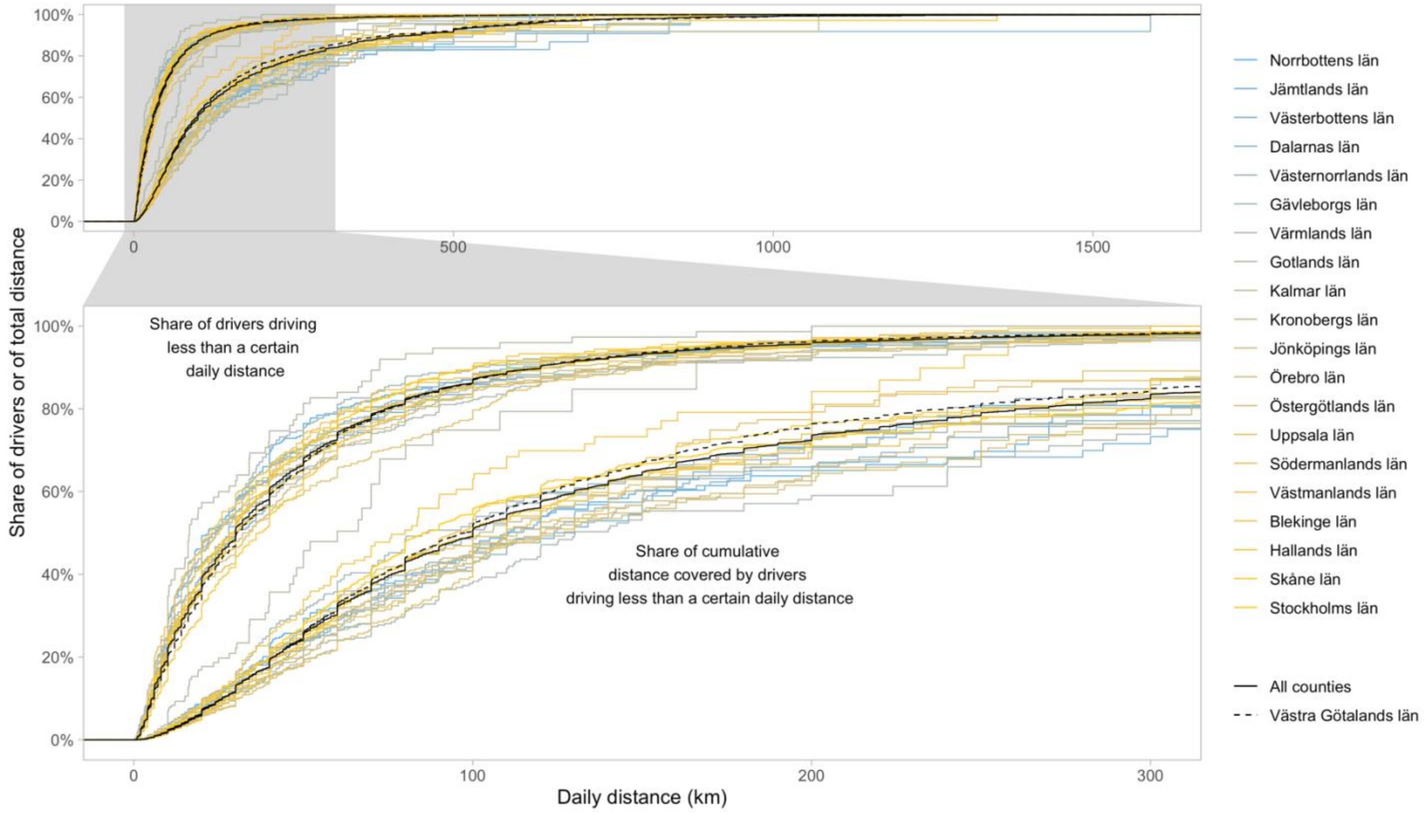

Figure SI 1: Cumulative share of individual drivers that drive less than a certain daily distance and cumulative share of distance covered by those drivers, for Swedish counties (*län*) ranked by population density (blue – low, and yellow – high).

1.3 Carbon footprint estimations of road and charging infrastructure

The emission factors for road construction, maintenance and rehabilitation are assumed to be 268 ton CO_2 per km (highway lane) for traditional roads and 590 ton CO_2 per km for roads with inductive ERS, including increased maintenance and rehabilitation due to Swedish winter conditions[10]. This estimate has been criticized for not including the components necessary for achieving inductive charging[11], but only the materials used in the charger (i.e., copper used in the coil for the case of inductive charging). Marmiroli et al.[11] estimate the emissions related to charging components to be 100 ton CO_{2e} per km, while Balieu et al.[10] estimate 19 ton CO_2 per km for producing the copper used in the charging system. Hence, the emission factor for constructing an inductive ERS may be slightly underestimated by Balieu et al.[10]. Their estimates are nevertheless deemed the most suitable for this study since they are developed for the Swedish case.

The emission factors estimated by Balieu et al.[10] are considered to be in line with current best available technologies and would decrease if additional emissions abatement efforts would be implemented. Since road construction is mainly supplied by domestic producers and additional policies are foreseen to achieve the net-zero target for Swedish territorial emissions by 2045[12], potential emissions abatement in road construction supply chains in Sweden needs to be taken into account when estimating the carbon footprint for future construction of an ERS. Karlsson et al.[13] estimate the emissions abatement potential for Swedish road construction supply chains to 65% by 2030 as compared to current best available technologies in a scenario that analyzes the pathway to reaching net-zero by 2045. Applying this emissions

abatement potential to the emission factors estimated by Balieu et al.[10] results in emission factors of 95 ton CO_2 per km and 209 ton CO_2 per km for traditional roads and roads with inductive ERS, respectively. Note that these estimates are used for both the stated policies and sustainable development pathways since the Swedish climate policy framework is deemed to fulfil the requirements of both being stated polices and in line with the sustainable development scenario goal.

The emission factors for stationary charging infrastructure are estimated based on the raw material demand for different types of stationary chargers provided by Zhang et al.[14] combined with the estimated emissions along those raw material supply chains as estimated by Morfeldt et al.[15] . The resulting emission factors are 0.5, 0.06 and 0.03 ton CO_2 per charger deployed in 2020 for public fast (direct current), public slow (alternating current) and private slow chargers, respectively. Stationary chargers are assumed to be manufactured in global markets. Hence, these emission factors decrease as raw material production decarbonizes in the Sustainable Development scenario and remain unchanged in the Stated Policies scenario.

Note that the additional impact of the on-board wireless charger is considered to be negligible since only circa 5% of the carbon footprint (excl. battery) is estimated to be related to the electronics needed in a regular battery electric vehicles, see GREET® 2 – Version 2019[16].

2 Detailed results

2.1 Tailpipe and fuel cycle emissions

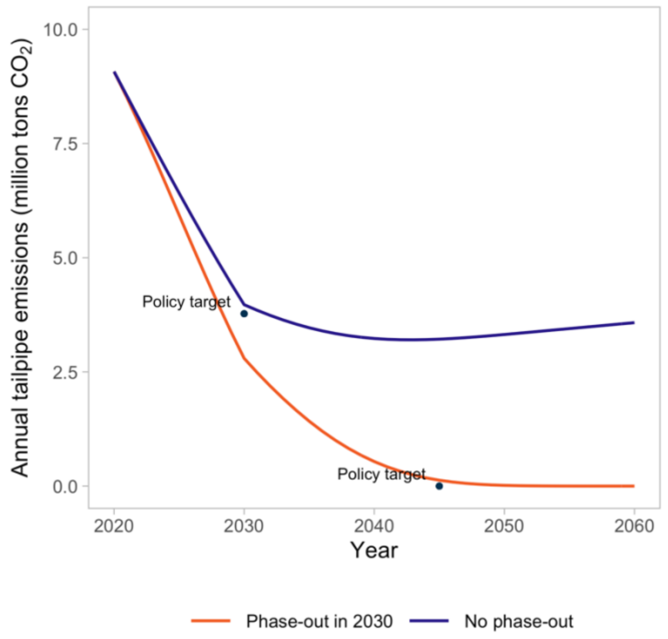

Figure SI 2: Annual tailpipe emissions for Swedish passenger car travel. Note that this emission pathway is equal for all scenarios analyzed.

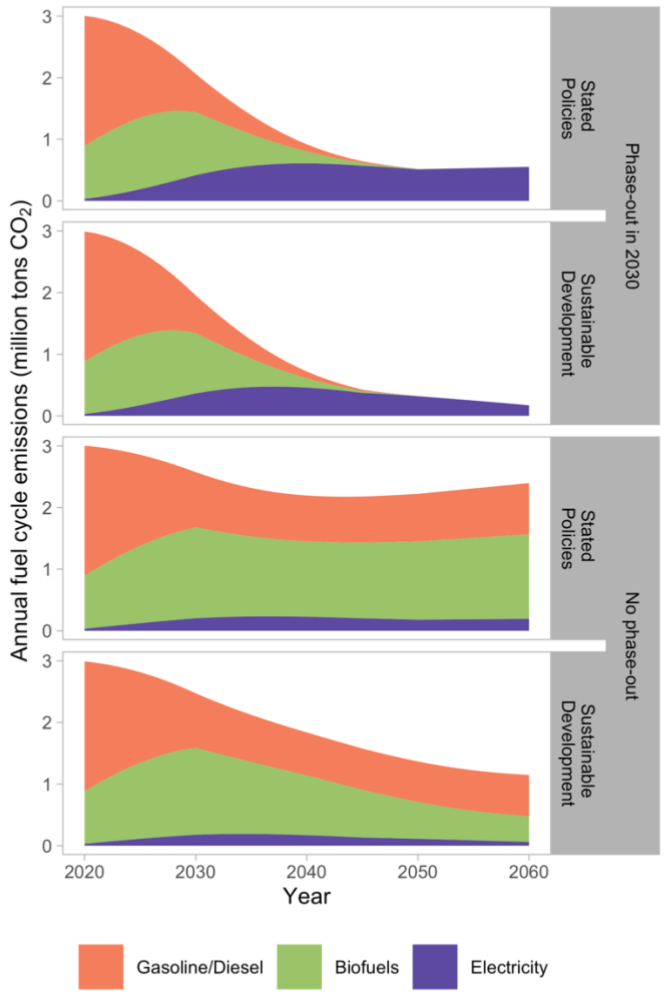

Figure SI 3: Annual fuel cycle emissions for Swedish passenger car travel. Note that these emissions depend on the assumed climate change mitigation pathway of global manufacturing

2.2 Vehicle fleet dynamics

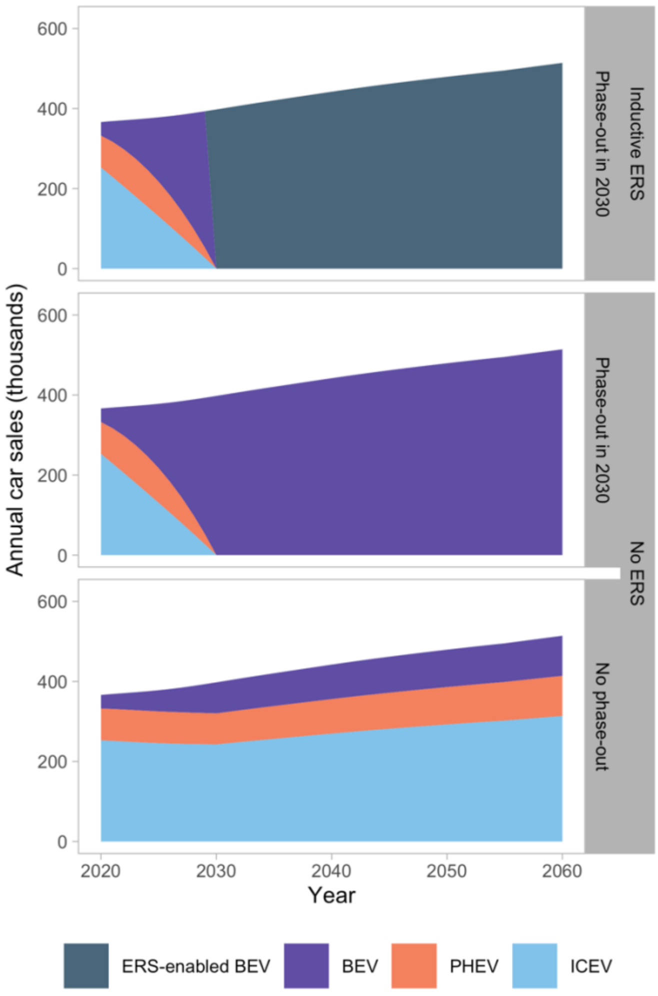

Figure SI 4: Annual sales of Swedish passenger cars for the analyzed drivetrains under the assumption of a ban on ICEs in 2030, depending on implementation of ERS.

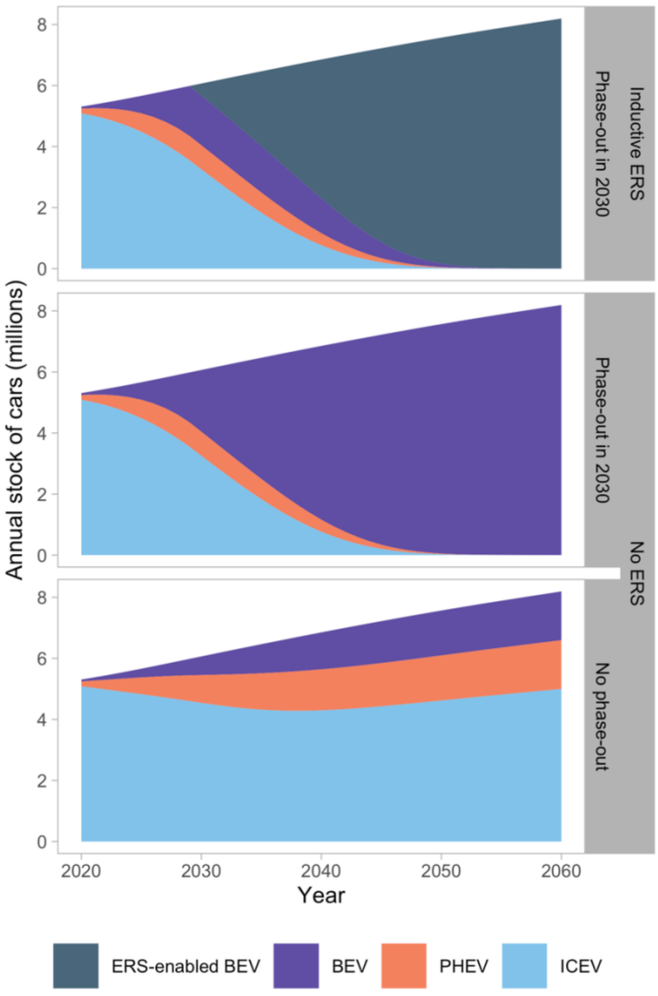

Figure SI 5: Annual stock of cars (i.e., cars available in the fleet) available to supply Swedish passenger car travel demand for the analyzed drivetrains, depending on implementation of ERS.

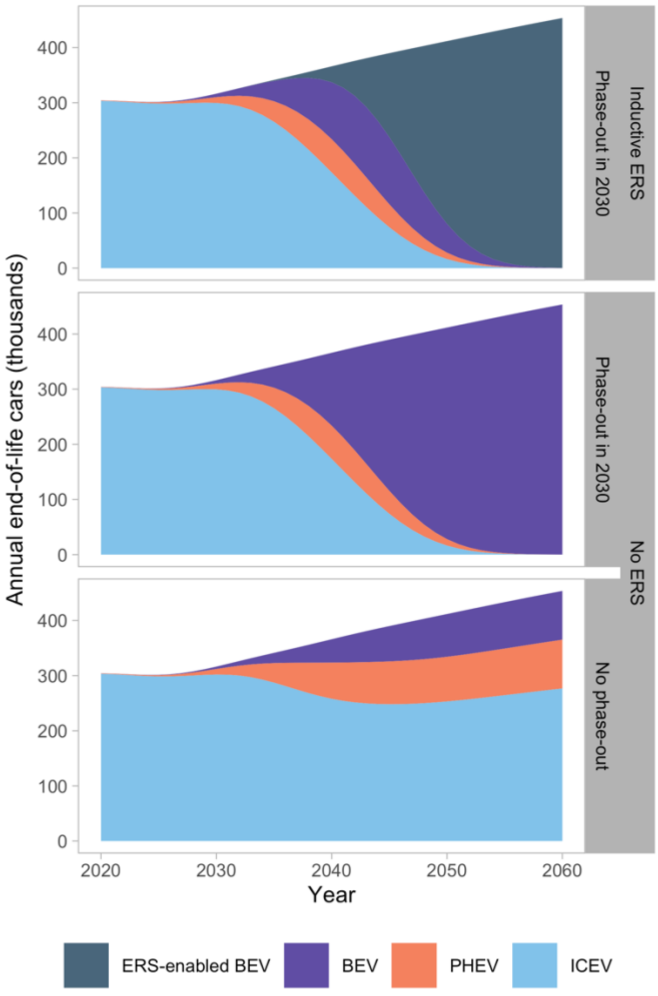

Figure SI 6: Annual number of Swedish passenger cars reaching end-of-life for the analyzed drivetrains, depending on implementation of ERS.

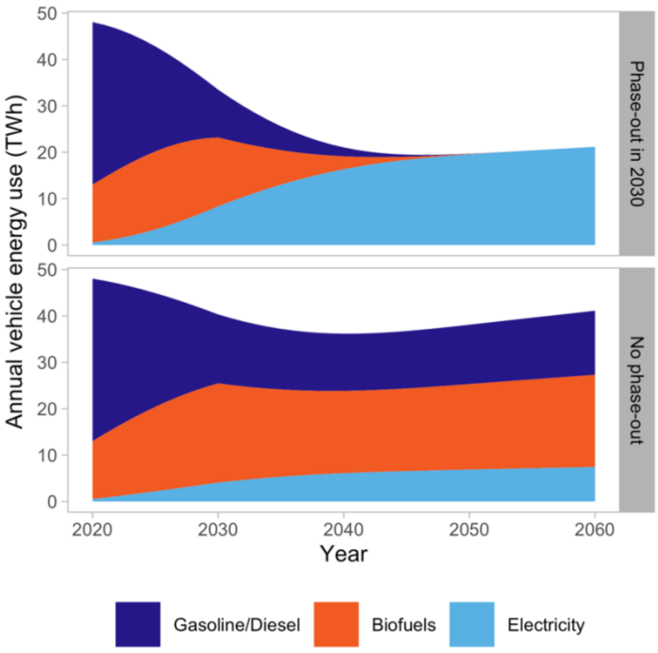

Figure SI 7: Annual vehicle energy use per energy carrier for Swedish passenger car travel. Note that these pathways are equal for all scenarios analyzed meaning that vehicle energy use is assumed to be equal regardless of ERS implementation.

2.3 Sensitivity analysis – ERS on 100% of E&N roads and for home-only stationary charging

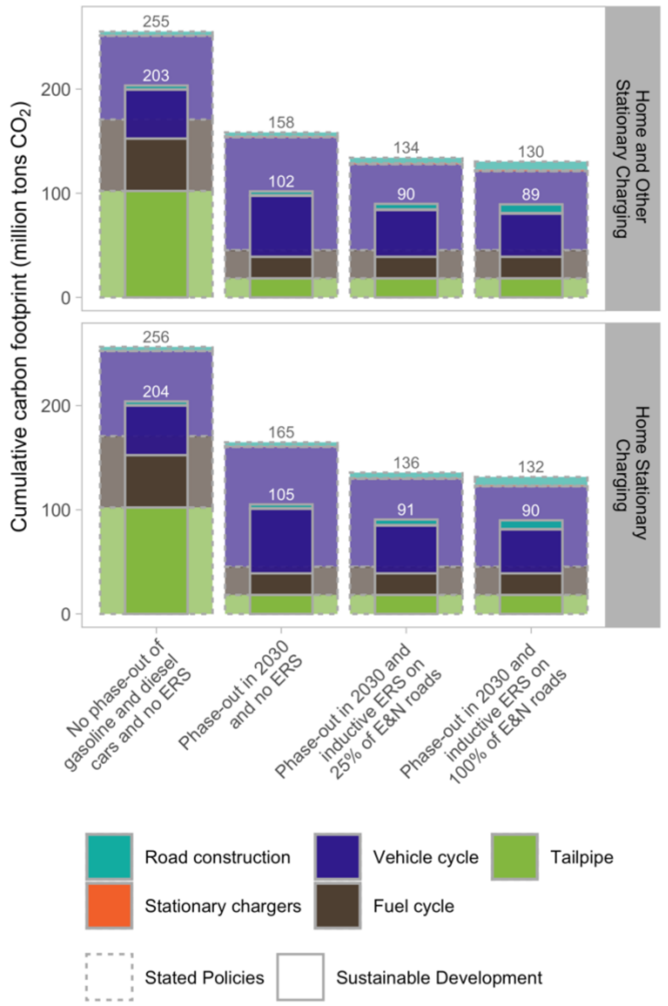

Figure SI 8: Cumulative carbon footprint of Swedish passenger car travel over the lifetime of an ERS for all scenarios compared to ERS placement on 100% of E&N roads, modelled for the period of 2030 – 2060. The ban is for gasoline and diesel cars.

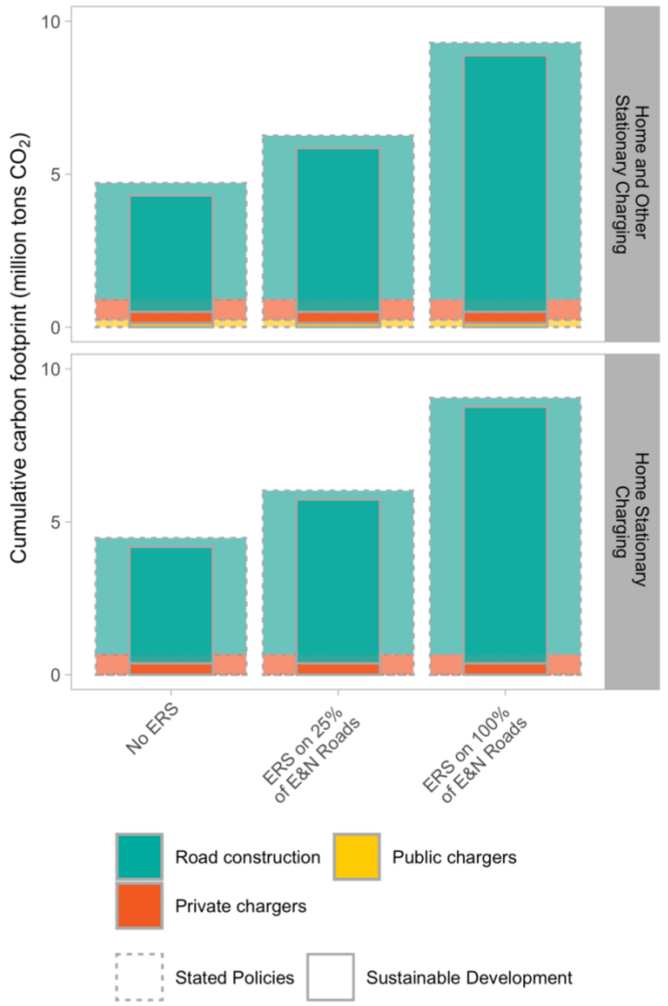

Figure SI 9: Cumulative carbon footprint for infrastructure construction and maintenance for all scenarios compared to ERS placement on 100% of E&N roads, modelled for the period of 2030 – 2060.

2.4 Sensitivity analysis – Vehicle and battery lifetime

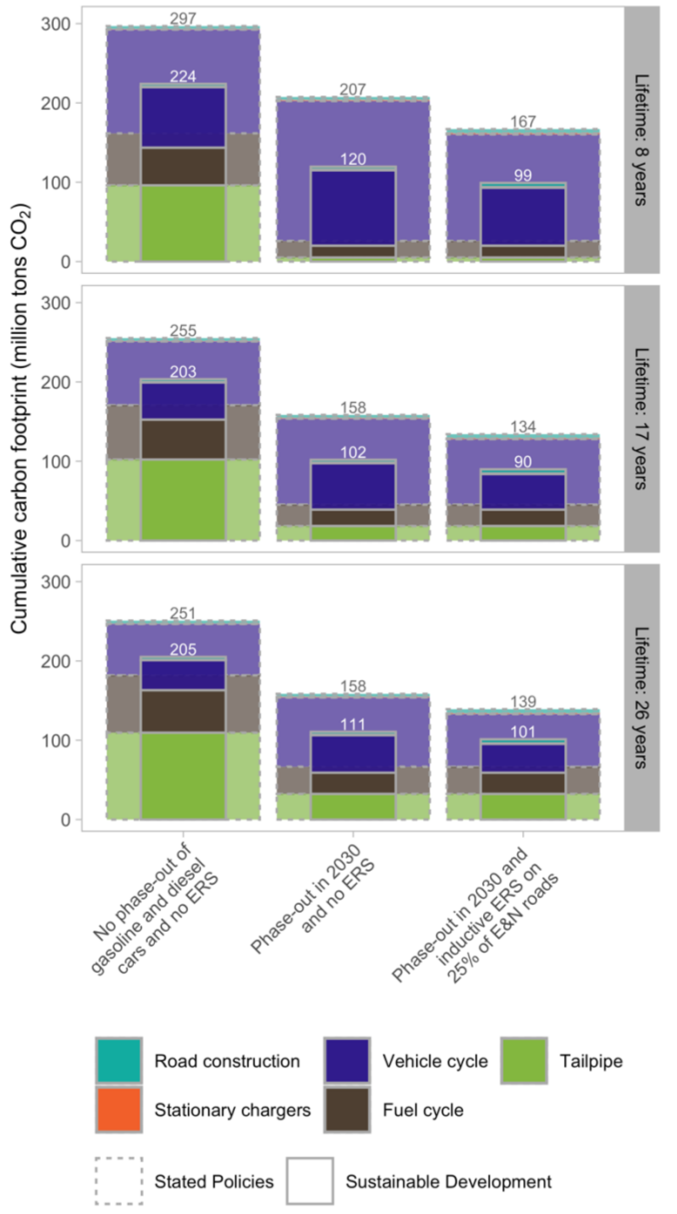

Figure SI 10: Cumulative carbon footprint of Swedish passenger car travel over the lifetime of an ERS for all scenarios, modelled for the period of 2030 – 2060. The ban is for gasoline and diesel cars. Sensitivity cases with varying assumed vehicle and battery lifetime. ERS placement on 25% of E&N roads, home and other stationary charging, transfer power of 2e and fixed battery sizes (5 kWh-steps).

The battery lifetime is assumed to be equal to the vehicle lifetime in this study, based on a statistical analysis of Swedish vehicle retirements, see SI 1.1. However, battery life for electric vehicles is still uncertain and will depend on the future progress in further developing battery technologies. Hence, a sensitivity analysis has been included to test how the results are influenced by this assumption. The sensitivity cases analyzed (8 years, 17 years and 25 years lifetime) are based on the 5th percentile, the mean and the 95th percentile of Swedish vehicle retirements, respectively. 8 years of lifetime also corresponds to the warranty for most vehicle manufacturers[17] and is therefore considered a realistic lower estimate. Vehicle and battery lifetimes are still considered equal.

The results show an expected, significant increase in cumulative vehicle cycle emissions for short vehicle lifetimes due to the need of replacing vehicles a little over 2 times as often as when vehicle lifetimes are 17 years, see Figure SI 10. The opposite effect can be seen for longer lifetimes. The vehicle lifetime also affects the impact of a phase-out of internal combustion engines in 2030, where a longer lifetime means that internal combustion engines will stay in the fleet for longer and supply parts of the travel demand passed the year 2050. As a result, the cumulative tailpipe and fuel cycle emissions are slightly higher for longer lifetimes.

Nevertheless, the benefits of ERS are still significant in all three sensitivity cases. The reduction in cumulative emissions from installing ERS on 25% of European and National roads is 9-19 MtCO_2 for the shorter vehicle lifetime and 20-40 MtCO_2 for the longer lifetime, which can be compared to 12-24 MtCO_2 for the main case.

2.5 Sensitivity analysis – ERS transfer power

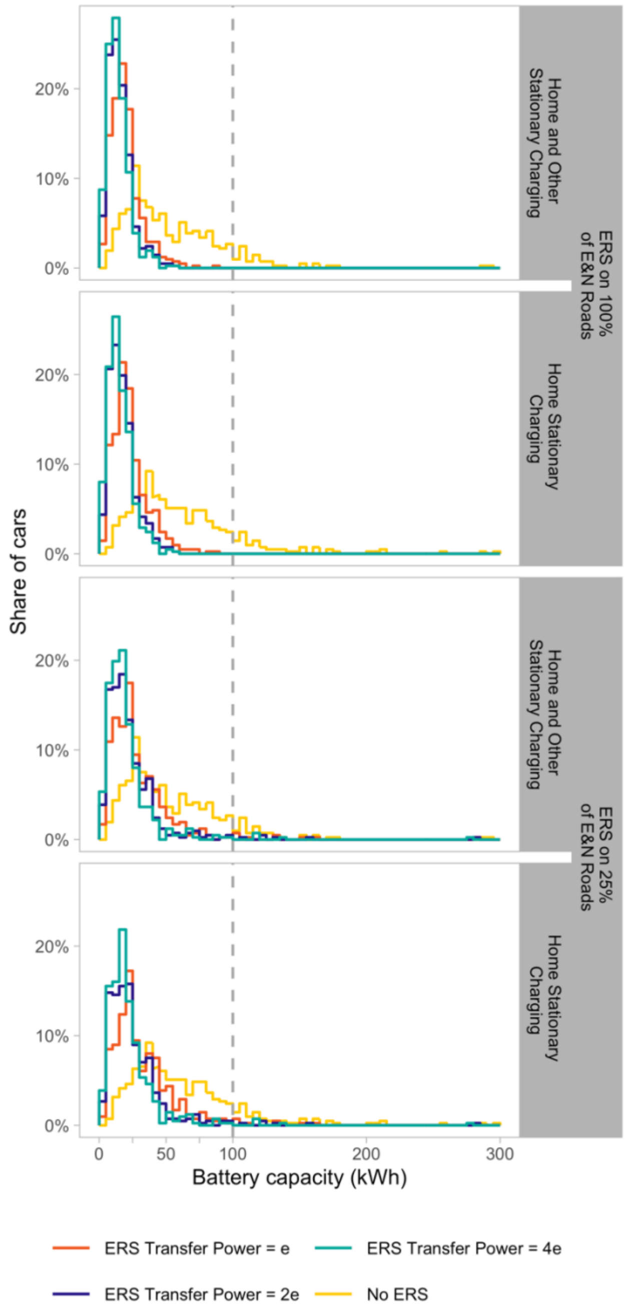

Figure SI 11: Share of cars with specific battery capacities, assuming post-2030 average specific energy use. Sensitivity cases with varying transfer power of 1e – 4e.

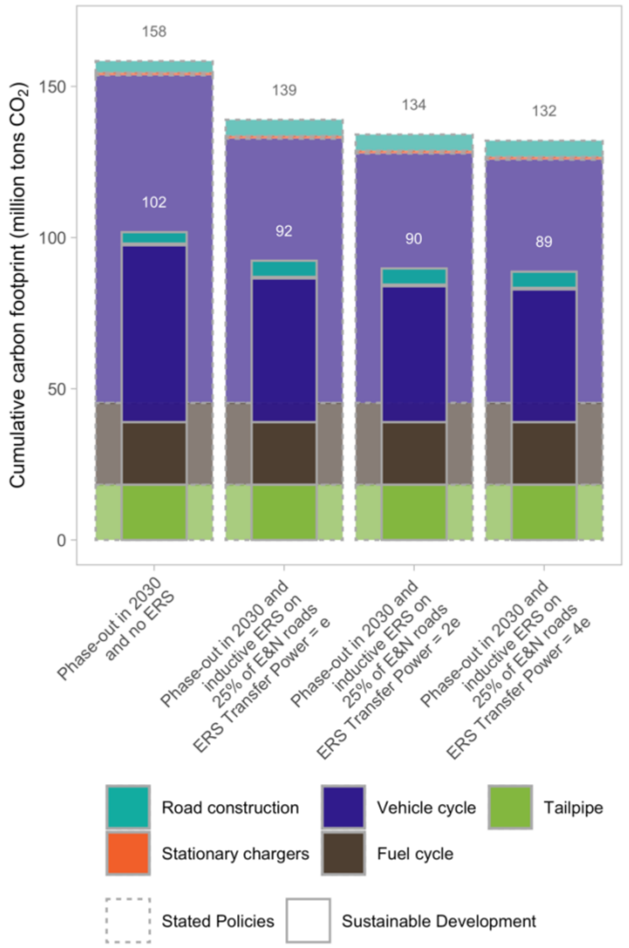

Figure SI 12: Cumulative carbon footprint of Swedish passenger car travel over the lifetime of an ERS for all scenarios, modelled for the period of 2030 – 2060. The ban is for gasoline and diesel cars. Sensitivity cases with varying transfer power of 1e – 4e. ERS placement on 25% of E&N roads, home and other stationary charging and fixed battery sizes (5 kWh-steps).

The transfer power of an ERS is uncertain. The transfer power sensitivity analysis tests the significance on the results of the assumed transfer power, varied between 1*e* and 4*e*. The results show that the potential for battery downsizing when implementing an ERS is high even when only matching the transfer power with the vehicle energy use (i.e., 1*e*), resulting in a median reduction in battery capacity of 49%, see Figure SI 11. The additional benefits of implementing an ERS transferring power above the vehicle energy use are incremental (59% for 2e and 64% for 4e, both compared to median battery capacities without ERS). The sensitivity analysis shows that the avoided emissions would be lower but still significant if transfer power equal to the specific energy use of the vehicle is assumed (9-19 million tons of CO_2 for 1e compared to 12-24 million tons for 2e), see Figure SI 12. Hence, the conclusions of the study remain valid as long as the

technology at least matches the vehicle energy use even though the technical specifications of an ERS are still uncertain, including its transferring power.

2.6 Sensitivity analysis – assumptions on battery sizes available in the market

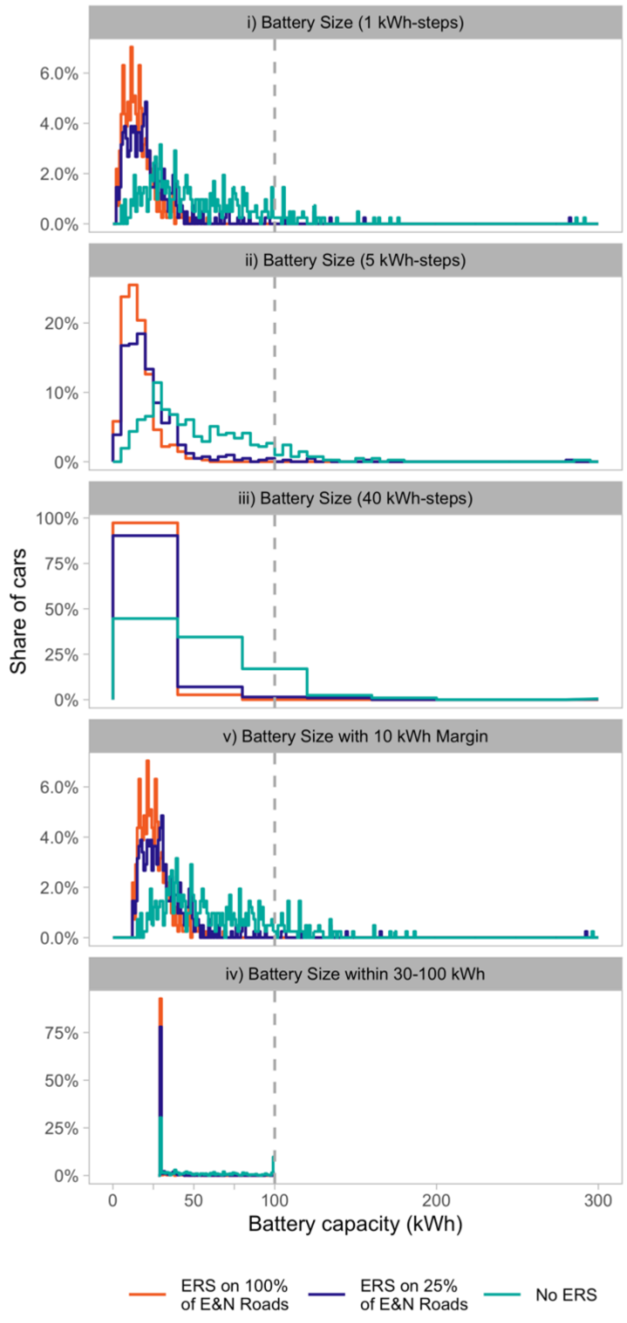

Figure SI 13: Share of cars with specific battery capacities, assuming post-2030 average specific energy use. Sensitivity cases with varying battery sizes available in the market. ERS transfer power of 2e.

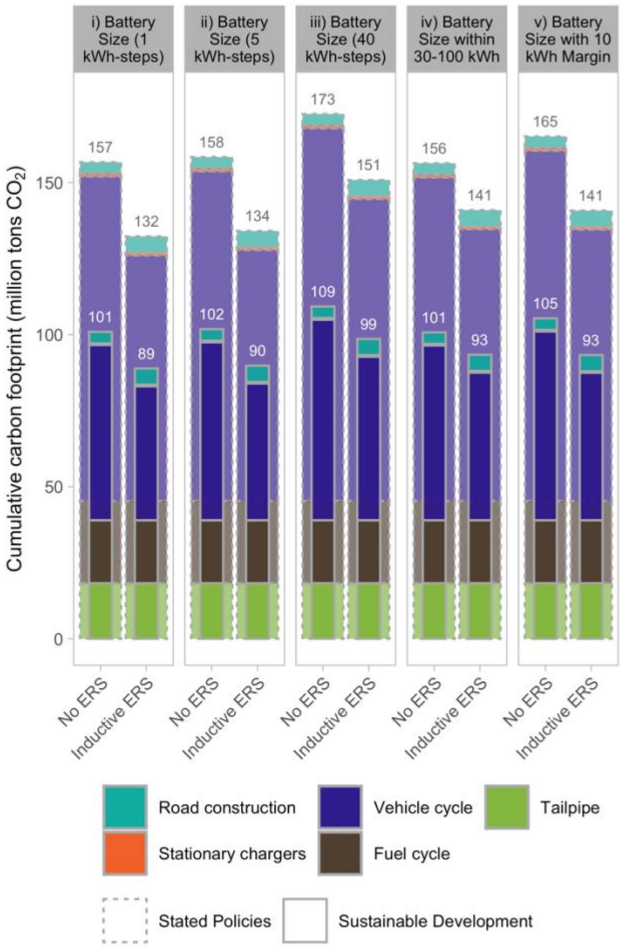

Figure SI 14: Cumulative carbon footprint of Swedish passenger car travel over the lifetime of an ERS for all scenarios, modelled for the period of 2030 – 2060. The ban is for gasoline and diesel cars. Sensitivity cases with varying battery sizes available in the market. ERS placement on 25% of E&N roads, home and other stationary charging and ERS transfer power of 2e.

The sensitivity analysis considers four cases in comparison to our main case (ii): fixed battery sizes of 1 kWh increments (i), fixed battery sizes of 40 kWh increments (iii), battery sizes with 10 kWh margin (iv) and fixed battery sizes within 30-100 kWh (v), see Figure SI 13. Available battery size options for users to choose from may be limited in the future, which is tested in the sensitivity cases with fixed sizes of 1, 5 or 40 kWh (cases i-iii). Range anxiety may discourage users from buying an optimal but small battery, which is tested in the sensitivity case that assumes a margin of 10 kWh (case iv), equivalent to about 50 km, on top of the optimal size suggested by the model. Finally, limited battery supply and good

access to fast chargers for users with long-range needs may limit the upper boundary for battery sizes. Also, range anxiety among users could limit lower end for battery sizes. Both limits are tested in the last sensitivity case where only battery sizes within 30-100 kWh are considered (case v). The results show that the case of fixed battery sizes of 40 kWh increments results slightly lower emissions reductions in the cumulative carbon footprint (11-22 million tons of CO_2) and that restricting the range of sizes to between 30-100 kWh significantly reduces those emissions reductions (7-15 million tons of CO_2), see Figure SI 14. The impact on the emissions reduction in the cumulative carbon footprint is insignificant for all other cases. The reductions in the cumulative carbon footprint from implementing an ERS are in these cases equal to the main case – 12-24 million tons of CO_2 over the 30-year period. Analyses of varying the adoption rate of batteries for these cases reveal that also those conclusions are robust – that most of the benefit of the ERS is realized already when 50% of drivers have adopted the ERS-enabled BEV, see SI 2.7.

2.7 Sensitivity analysis – higher emissions in road construction and maintenance

For road construction and maintenance related emissions, the uncertainty of emission abatement efforts in global manufacturing is not considered since road construction and production of the materials used are assumed to be occur domestically. Nevertheless, a sensitivity analysis is needed to highlight the impact on the results if Swedish road construction does not decarbonize, effectively missing the domestic policy target. To illustrate such a scenario, the emission factors by Balieu et al.[10] have been applied as is, without the reduction explained in section 1.2 of the Supplementary Materials. Additional emissions accounting for the risk of a higher carbon footprint for charging components in the ERS are also included, also discussed in section SI 1.2.

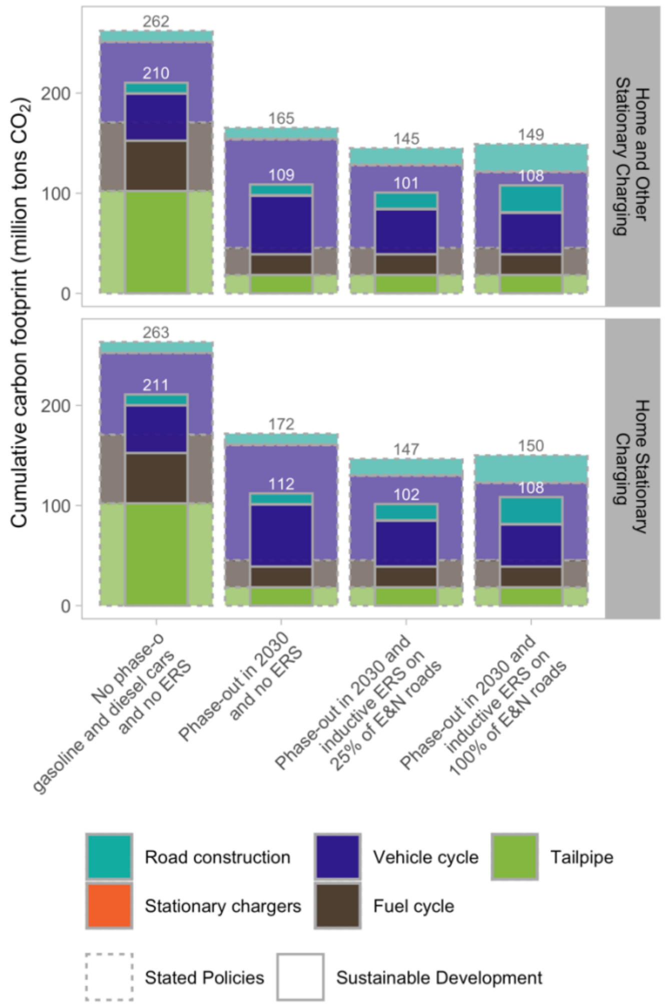

Figure SI 15: Cumulative carbon footprint over the lifetime of an ERS, modelled for the period of 2030 – 2060. The ban is for gasoline and diesel cars. Sensitivity case for higher emissions in road construction and maintenance.

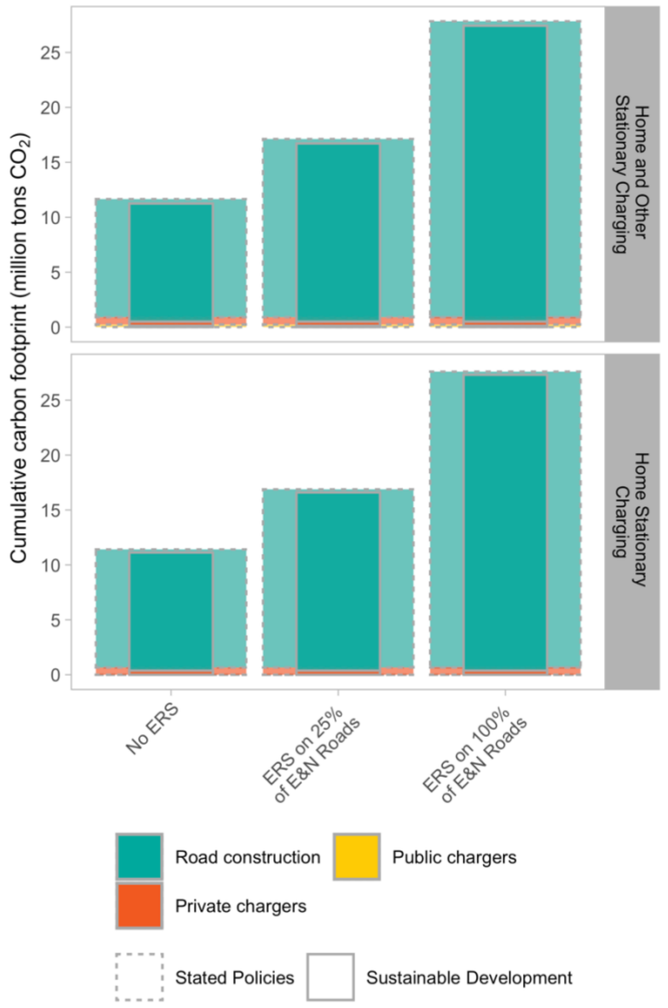

Figure SI 16: Cumulative carbon footprint for charging and road infrastructure over the lifetime of an ERS, modelled for the period of 2030 – 2060. Sensitivity case for higher emissions in road construction and maintenance.

In such a scenario, the carbon footprint of road construction and maintenance would increase from the higher level of 10.7 $MtCO_2$ without an ERS to 16.2 and 27.0 $MtCO_2$ for implementation on 25% and 100% of E&N, respectively, see Figure SI 16. This means that the emission abatement potential of implementing an ERS for the total cumulative carbon footprint in this case is slightly lower (8-20 $MtCO_2$ for an ERS on 25% of E&N roads, as compared to 12-24 $MtCO_2$ for the scenario presented in section 3.1 of the main text), see Figure SI 15. Furthermore, extending the ERS coverage to 100% of E&N roads would in this case increase the cumulative carbon footprint as compared to the 25% case.

2.8 Sensitivity analysis – importance of the adoption rate of ERS-enabled BEVs

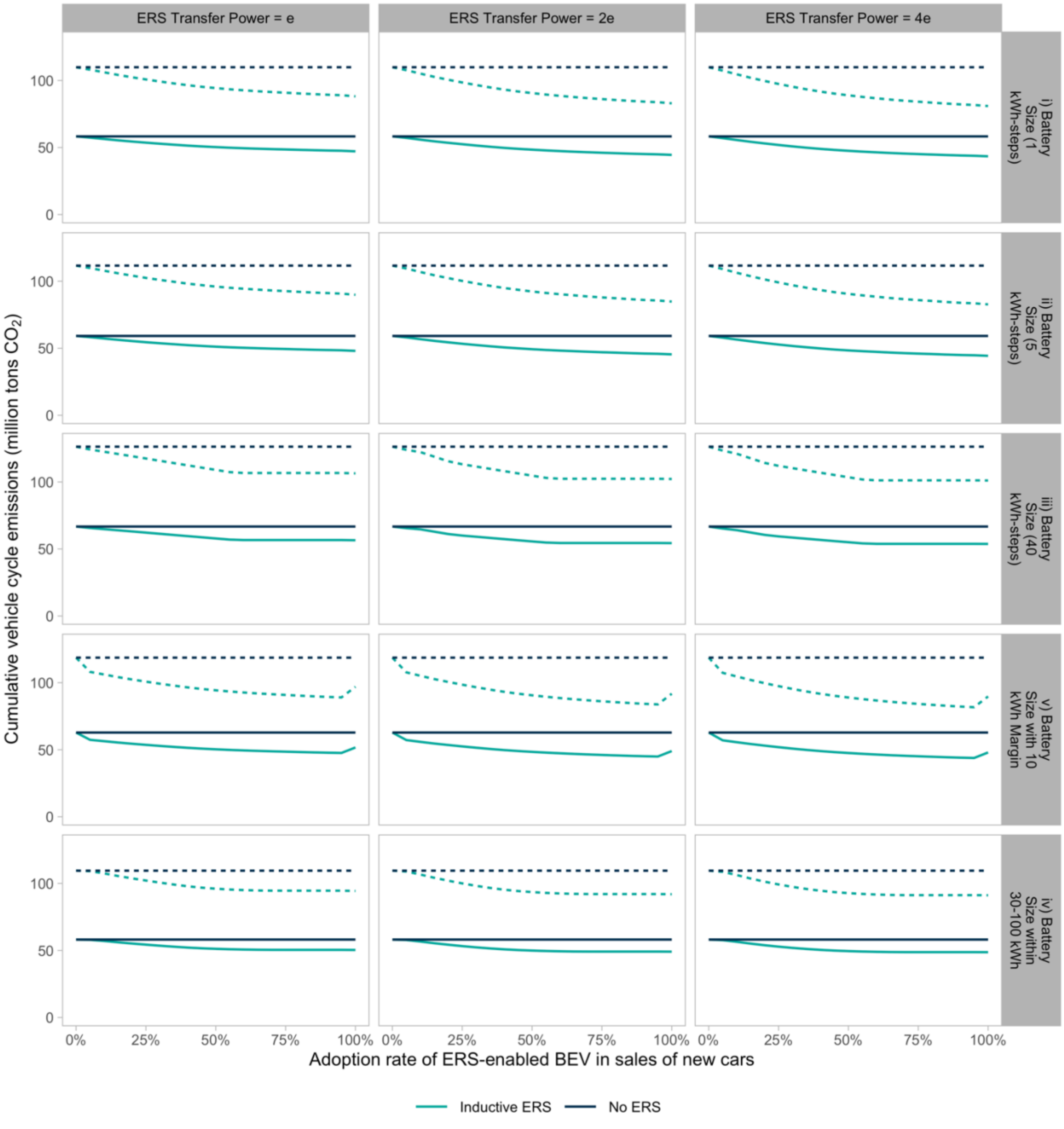

Figure SI 17: Cumulative vehicle cycle emissions for the period 2030-2060 depending on different adoption rates of ERS-enabled BEV as new car sales. Sensitivity cases with varying transfer power of 1e – 4e (columns) and varying battery sizes available in the market (rows).

References

(1) Transport Analysis. *Detailed Excerpt on Scrappage of Vehicles from the Statistics “Fordon På Väg” (Road Vehicles) for the Years 2014-2018*; Stockholm, Sweden, 2020; https://www.trafa.se/vagtrafik/fordon/.

(2) Jeong, S.; Jang, Y. J.; Kum, D.; Lee, M. S. Charging Automation for Electric Vehicles: Is a Smaller Battery Good for the Wireless Charging Electric Vehicles? *IEEE Transactions on Automation Science and Engineering* **2019**, *16* (1), 486–497. https://doi.org/10.1109/TASE.2018.2827954.

(3) Limb, B. J.; Asher, Z. D.; Bradley, T. H.; Sproul, E.; Trinko, D. A.; Crabb, B.; Zane, R.; Quinn, J. C. Economic Viability and Environmental Impact of In-Motion Wireless Power Transfer. *IEEE Transactions on Transportation Electrification* **2019**, *5* (1), 135–146. https://doi.org/10.1109/TTE.2018.2876067.

(4) De Gennaro, M.; Paffumi, E.; Martini, G.; Giallonardo, A.; Pedroso, S.; Loiselle-Lapointe, A. A Case Study to Predict the Capacity Fade of the Battery of Electrified Vehicles in Real-World Use Conditions. *Case Studies on Transport Policy* **2020**, *8* (2), 517–534. https://doi.org/10.1016/j.cstp.2019.11.005.

(5) Bi, Z.; Keoleian, G. A.; Lin, Z.; Moore, M. R.; Chen, K.; Song, L.; Zhao, Z. Life Cycle Assessment and Tempo-Spatial Optimization of Deploying Dynamic Wireless Charging Technology for Electric Cars. *Transportation Research Part C: Emerging Technologies* **2019**, *100* (May 2018), 53–67. https://doi.org/10.1016/j.trc.2019.01.002.

(6) Yang, X. G.; Liu, T.; Wang, C. Y. Thermally Modulated Lithium Iron Phosphate Batteries for Mass-Market Electric Vehicles. *Nature Energy* **2021**, *6* (February). https://doi.org/10.1038/s41560-020-00757-7.

(7) Karlsson, S. *The Swedish Car Movement Data Project - Final Report, PRT Report 2013:1, Rev 2*; Chalmers University of Technology: Gothenburg, Sweden, 2013; https://research.chalmers.se/en/publication/187380.

(8) Transport Analysis. Travel Survey https://www.trafa.se/en/travel-survey/travel-survey/ (accessed Mar 16, 2021).

(9) Statistics Sweden. Population density per sq. km, population and land area by region and sex. Year 1991 - 2020 https://scb.se/BE0101 (accessed Mar 8, 2021).

(10) Balieu, R.; Chen, F.; Kringos, N. Life Cycle Sustainability Assessment of Electrified Road Systems. *Road Materials and Pavement Design* **2019**, *20* (sup1), S19–S33. https://doi.org/10.1080/14680629.2019.1588771.

(11) Marmiroli, B.; Dotelli, G.; Spessa, E. Life Cycle Assessment of an On-Road Dynamic Charging Infrastructure. *Applied Sciences* **2019**, *9* (15), 3117. https://doi.org/10.3390/app9153117.

(12) Swedish Government. *Regeringens Proposition 2019/20:65 En Samlad Politik För Klimatet – Klimatpolitisk Handlingsplan (Governmental Bill 2019/20:65 Joint Policy for Climate Change - Climate Policy Action Plan)*; 2020; https://www.regeringen.se/rattsliga-dokument/proposition/2019/12/prop.-20192065/.

(13) Karlsson, I.; Rootzén, J.; Johnsson, F. Reaching Net-Zero Carbon Emissions in Construction Supply Chains – Analysis of a Swedish Road Construction Project. *Renewable and Sustainable Energy Reviews* **2020**, *120*, 109651. https://doi.org/10.1016/j.rser.2019.109651.

(14) Zhang, Z.; Sun, X.; Ding, N.; Yang, J. Life Cycle Environmental Assessment of Charging Infrastructure for Electric Vehicles in China. *Journal of Cleaner Production* **2019**, *227*, 932–941. https://doi.org/10.1016/j.jclepro.2019.04.167.

(15) Morfeldt, J.; Davidsson Kurland, S.; Johansson, D. J. A. Carbon Footprint Impacts of Banning Cars with Internal Combustion Engines. *Transportation Research Part D: Transport and Environment* **2021**, *95*, 102807. https://doi.org/10.1016/j.trd.2021.102807.

(16) Argonne National Laboratory. The Greenhouse Gases, Regulated Emissions, and Energy Use in Transportation (GREET®) Model - GREET 2, Version 2019 https://greet.es.anl.gov/ (accessed Jan 9, 2020).

(17) Murphy, L. Which Electric Car Has the Best Battery Warrenty? *DRIVE*. July 14, 2020.
